# Quantum linear solvers for scientific computing: a comparison of VQLS, HHL and quantum annealing on time-fractional diffusion problems

**DOI:** 10.1038/s41598-026-40910-y

**Published:** 2026-02-23

**Authors:** Amir Hossein Salehi Shayegan

**Affiliations:** Faculty of Mathematics, K. N. Toosi University of Technology, Tehran, Iran

**Keywords:** Time-fractional diffusion equation, Anomalous transport phenomena, VQLS, HHL, Quantum annealing, QUBO formulation, Engineering, Mathematics and computing, Physics

## Abstract

Time-fractional diffusion equations have emerged as powerful models for describing anomalous transport phenomena in physics, biology and engineering. To address the computational challenges arising from their non-local operators, we employ the WEB-spline finite element method, which provides a flexible and accurate discretization framework. The resulting linear system of equations are then explored in the context of quantum computing. Specifically, we investigate three prominent quantum linear solvers: the variational quantum linear solver (VQLS), the Harrow-Hassidim-Lloyd (HHL) algorithm and quantum annealing (QA). VQLS leverages hybrid variational techniques and shallow circuits, making it well-suited for noisy intermediate-scale quantum (NISQ) devices, while HHL offers a theoretically exponential speedup for sparse systems but requires deep fault-tolerant circuits. QA, in contrast, reformulates the problem into a quadratic unconstrained binary optimization (QUBO) instance, enabling approximate solutions through energy minimization on specialized hardware. We present a comparative analysis in terms of circuit depth, noise resilience, scalability and solution extraction, including the role of quantum state tomography in reconstructing classical information. Numerical experiments on a time-fractional diffusion problem highlight the complementary strengths and limitations of each method. This study bridges advanced numerical discretization with emerging quantum algorithms, providing insights into the feasibility and future potential of quantum-enhanced solvers for fractional partial differential equations.

## Introduction

Fractional partial differential equations (FPDEs), particularly time-fractional diffusion equations, have become essential tools for modeling anomalous transport and memory-dependent phenomena in physics, biology and engineering^1–4^. Unlike classical diffusion equations, time-fractional models incorporate non-local temporal derivatives, which capture sub-diffusive or super-diffusive behavior and provide more accurate descriptions of real-world processes. However, this non-locality introduces significant computational challenges, especially when dealing with large-scale systems or fine temporal discretizations. To overcome these challenges, advanced numerical techniques are required. The WEB-spline finite element method offers a flexible and accurate framework for spatial discretization, while efficiently handling the complexities of fractional derivatives^5,6^. The combination of WEB-spline finite element method with appropriate temporal discretization schemes allows for high-accuracy approximations of the solution while maintaining stability and convergence properties. After spatial and temporal discretizations, the resulting linear systems are often large and sparse, motivating the exploration of emerging quantum computational techniques. Quantum algorithms for linear systems, such as VQLS, HHL algorithm and quantum annealing provide new avenues to solve these problems efficiently under certain conditions. VQLS leverages hybrid variational approaches and shallow circuits suitable for NISQ devices (see Appendix ??), HHL offers exponential speedup for sparse, well-conditioned matrices but requires fault-tolerant hardware and sophisticated measurement protocols, while QA reformulates the problem into a QUBO form and outputs classical solutions directly^7–10^.

The application of quantum algorithms to FPDEs is an emerging area of research, motivated by the high computational cost of classical methods for time and space-fractional problems. The HHL algorithm has been explored for solving linear systems arising from the discretization of FPDEs, providing theoretical exponential speedup for sparse and well-conditioned matrices^8,11,12^. Variational approaches, such as VQLS, have been adapted to handle the large, sparse systems resulting from fractional discretizations while maintaining robustness on NISQ devices^13^. Quantum annealing has also been investigated, where FPDE discretizations are reformulated as QUBO problems, enabling approximate solutions through energy minimization on quantum annealers^10,14^. Recent studies highlight that while HHL can theoretically yield exact solutions, it requires deep, fault-tolerant circuits and careful quantum state tomography to extract classical information, whereas VQLS offers flexible ansatz-dependent encoding and QA provides hardware-efficient approximate solutions with direct classical outputs. Despite these promising developments, practical implementations are still limited by the system size, sparsity and measurement constraints, making the combination of advanced numerical discretizations, such as WEB-spline finite element method, with quantum solvers a crucial research direction. Also for recognizing the close methodological overlap (encoding, parametrized circuits, hybrid optimization and scalability), one can refer to^15,16^.

The main contributions of this work are the development of a WEB-spline finite element method framework for accurately discretizing time-fractional diffusion problems with stability and high-order accuracy, the implementation and comparative analysis of three leading quantum solvers, VQLS, HHL and quantum annealing, on the resulting linear system of equations, the evaluation of these methods in terms of circuit depth, sparsity requirements, noise resilience and solution extraction including the role of quantum state tomography and the presentation of numerical results demonstrating the practical feasibility and complementary strengths of these quantum algorithms for solving fractional partial differential equations. This study bridges advanced numerical discretization techniques with state-of-the-art quantum algorithms, offering a comprehensive perspective on the potential and limitations of quantum-enhanced solvers for time-fractional diffusion problems.

This paper is organized as follows. In Section 2, we present the discretization of the time-fractional diffusion problem using a time discretization scheme and the WEB-spline finite element method. Section 3 introduces the variational quantum linear solver for solving the resulting linear system of equations. In Section 4, we describe the HHL quantum algorithm for linear system solution, followed by Section 5, which discusses quantum annealing approaches for the same task. Finally, Section 6 concludes the paper with a comparative analysis of VQLS, HHL, and quantum annealing, highlighting their advantages, limitations and suitability for practical implementation.

## Discretization of the time-fractional diffusion problem

Consider the following time-fractional diffusion problem:

1 $$\begin{aligned} & ^CD_t^\alpha u({\textbf {x}},t) - \Delta u({\textbf {x}},t) = f({\textbf {x}},t),{\hspace{0.55542pt}} {\hspace{0.55542pt}} {\hspace{0.55542pt}} {\hspace{0.55542pt}} {\hspace{0.55542pt}} \,\,\,\,\,\,\,\,({\textbf {x}},t) \in {Q_T},\,\,\,\,\,\,\,\,\,\,\,\,\,\,\,\,\,\,\,\,\,\,\,\,\,\,\,\,\,\,\,\,\,\,\,\,\,\,\,\,\,\,\,\,\end{aligned}$$

2 $$\begin{aligned} & u({\textbf {x}},t) = 0, \,\,\,\,\,\,\,\,\,\,\,\,\,\,\,\,\,\,\,\,\,\,\,\,\,\,\,\,\,\,\,\,\,\,\,\,\,\,\,\,\,{\textbf {x}} \in \partial \Lambda ,\,\,t \in (0,T),\,\,\,\,\,\,\,\,\,\,\,\,\,\,\,\,\,\,\,\,\,\,\,\,\,\,\,\,\,\,\,\,\,\,\,\,\,\,\,\,\,\,\,\,\,\, \end{aligned}$$

3 $$\begin{aligned} & u({\textbf {x}},0) = \phi ({\textbf {x}}),\,\,\,\,\,\,\,\,\,\,\,\,\,\,\,\,\,\,\,\,\,\,\,\,\,\,\,\,\,\,\,\,\,\,\,\,\,\,\,\,\,\,\,\,\,\,\,\,\,\,\,\,\,\,\,\,\,\,\,\,\,\,\,\,{\textbf {x}} \in \Lambda ,\,\,\,\,\,\,\,\,\,\,\,\,\,\,\,\,\,\,\,\,\,\,\,\,\,\,\,\,\,\,\,\,\,\,\,\,\,\,\,\,\,\,\,\,\,\, \end{aligned}$$

where $\Lambda = \left\{ {{\textbf {x}} = \left( {x,y} \right) \left| {0 \le x \le 1,\,\,\,0 \le y \le 1 - x} \right. } \right\}$, $Q_T = \Lambda \times (0,T)$, and $0< \alpha < 1$. Here, ${}^{C}D_t^\alpha u({\textbf {x}},t)$ denotes the left-sided Caputo fractional derivative defined for $t \in (0,T]$ by

4 $$\begin{aligned} {}^{C}D_t^\alpha u({\textbf {x}},t) = \frac{1}{\Gamma (1 - \alpha )} \int _0^t \frac{\partial u}{\partial \tau }({\textbf {x}},\tau ) (t - \tau )^{-\alpha } \, d\tau . \end{aligned}$$

**Weak formulation:** The weak solution $u \in B^{\frac{\alpha }{2}}(Q_T)$ is defined as the function satisfying the integral identity:

$$\begin{aligned} \Pi (u,v) = F(v), \quad \forall v \in B^{\frac{\alpha }{2}}(Q_T), \end{aligned}$$

where the bilinear form $\Pi (\cdot ,\cdot )$ is given by:

5 $$\begin{aligned} \Pi (u,v) := \left( {}^{R}D_t^{\frac{\alpha }{2}} u, \; {}_t^{R} D^{\frac{\alpha }{2}} v \right) _{L_2(Q_T)} + (\nabla u, \nabla v)_{L_2(Q_T)}, \end{aligned}$$

and the linear functional $F(\cdot )$ is:

$$\begin{aligned} F(v) := (f, v)_{L_2(Q_T)}. \end{aligned}$$

Here, the function space

$$\begin{aligned} B^\alpha (Q_T) := H^\alpha \big ((0,T); L_2(\Lambda )\big ) \cap L_2\big ((0,T); H_0^1(\Lambda )\big ), \end{aligned}$$

is a Banach space equipped with the norm

$$\begin{aligned} \Vert v\Vert _{B^\alpha (Q_T)} = \left( \Vert v\Vert _{H^\alpha ((0,T); L_2(\Lambda ))}^2 + \Vert v\Vert _{L_2((0,T); H_0^1(\Lambda ))}^2 \right) ^{1/2}. \end{aligned}$$

It is known (see, e.g.,^17,18^) that if $0< \alpha < 1$, $f \in L_2(Q_T)$ and $\phi \in L_2(\Lambda )$, then the weak solution $u \in B^{\frac{\alpha }{2}}(Q_T)$ of the problem (1)–(3) exists and is unique. In the bilinear form (5), ${}^R D_t^\mu$ and ${}_t^R D^\mu$ denote the left and right Riemann-Liouville fractional derivatives of order $\mu$, respectively, defined for any integer *n* with $n-1 \le \mu < n$ by:

$$\begin{aligned} {}^R D_t^\mu w(t)&= \frac{1}{\Gamma (n - \mu )} \frac{d^n}{dt^n} \int _0^t \frac{w(\tau )}{(t - \tau )^{\mu - n + 1}} \, d\tau , \\ {}_t^R D^\mu w(t)&= \frac{(-1)^n}{\Gamma (n - \mu )} \frac{d^n}{dt^n} \int _t^T \frac{w(\tau )}{(\tau - t)^{\mu - n + 1}} \, d\tau . \end{aligned}$$

### Temporal discretization method

Let $t_k = k \tau$ for $k = 0, 1, \ldots , n$, where $\tau = T/n$ is the time step. The Caputo derivative $^C D_t^\alpha u({\textbf {x}},t)$ at $t_k$ is approximated as (see^19,20^):

6 $$\begin{aligned} ^C D_t^\alpha u({\textbf {x}}, t_k) \approx \frac{\tau ^{1-\alpha }}{\Gamma (2-\alpha )} \sum _{i=0}^{k-1} b_i \, \frac{u^{k-i} - u^{k-i-1}}{\tau }, \end{aligned}$$

with $b_i = (i-1)^{1-\alpha } - i^{1-\alpha }$ and $u^{k} = u({\textbf {x}}, t_k)$. Inserting (6) into (1)-(3) yields

$$\begin{aligned} - \Delta u^k + \frac{1}{\lambda } u^k = f_k + \frac{1}{\lambda } \sum _{i=1}^{k-1} \omega _i u^{k-i} + \frac{b_{k-1}}{\lambda } \phi (x) + \mathcal {R}, \end{aligned}$$

where $\lambda = \Gamma (2-\alpha ) \tau ^\alpha$, $\omega _i = b_{i-1} - b_i$, $f_k = f({\textbf {x}},t_k)$ and $|\mathcal {R}| \le C \tau ^{2-\alpha }$^20^. Neglecting $\mathcal {R}$, the discrete problem is derived as follows:

7 $$\begin{aligned} & - \Delta U^k + \frac{1}{\lambda }{U^k} = {f_k} + \frac{1}{\lambda }\sum \limits _{i = 1}^{k - 1} {{\omega _i}} {U^{k - i}} + \frac{{{b_{k - 1}}}}{\lambda }\phi ({\textbf {x}})\,\,\,\,\,\,\,\,\,\,\,\,\,\,\,{\textbf {x}} \in \Lambda ,\end{aligned}$$

8 $$\begin{aligned} & {U^k}({\textbf {x}}) = 0,\,\,\,\,\,\,\,\,\,\,\,\,\,\,\,\,\,\,\,\,\,\,\,\,\,\,\,\,\,\,\,\,\,\,\,\,\,\,\,\,\,\,\,\,\,\,\,\,\,\,\,\,\,\,\,\,\,\,\,\,\,\,\,\,\,\, \,\,\,\,\,\,\,\,\,\,\,\,\,\,\,\,\,\,\,\,\,\,\,\,\,\,\,\,\,\,\,\,\,\,\,{\textbf {x}} \in \partial \Lambda , \end{aligned}$$

where $U^{k}$ is the numerical solution at $t_k$.

### Spatial discretization method

Before discussing the spatial discretization approach for the problem (7)-(8), we provide a brief overview of the WEB-spline finite element method^5,6,21^.

#### WEB-spline finite element method

Let

$$\begin{aligned} b_n^{k,h}({\textbf {x}}) = b_n^{k,h}(x,y) = b_n^{{k_1},h}(x) \otimes b_n^{{k_2},h}(y),\quad k = ({k_1},{k_2}) \in K, \end{aligned}$$

where *h* is the spatial step, *K* is the set of nodes and

9 $$\begin{aligned} b_{n}^{k,h}(x) = b_n \left( \frac{x}{h} - k \right) , \end{aligned}$$

in which $b_n(x)$ is recursively defined by

10 $$\begin{aligned} b_n(x) = \frac{x}{n} b_{n-1}(x) + \frac{n+1 - x}{n} b_{n-1}(x-1), \quad n=2,3,\ldots , \end{aligned}$$

with the base case

11 $$\begin{aligned} b_1(x) = {\left\{ \begin{array}{ll} x, & 0 \le x \le 1, \\ 2 - x, & 1 \le x \le 2, \\ 0, & \text {otherwise}. \end{array}\right. } \end{aligned}$$

Here, $n$ denotes the degree of the B-spline. The support of the bivariate B-spline $b_{n}^{k,h}$ is:

12 $$\begin{aligned} \operatorname {supp}(b_{n}^{k,h}) = [k_1, k_1 + n + 1]h \times [k_2, k_2 + n + 1]h, \end{aligned}$$

where $h$ is the grid step in the $x$ and $y$ directions. The index set $K$ consists of all $k$ such that $\operatorname {supp}(b_{n}^{k,h}) \cap \Lambda \ne \emptyset$ for some ${\textbf {x}} = (x,y) \in \Lambda \subset \mathbb {R}^2$. We split $K$ into inner B-splines $I$ and outer B-splines $J$. If the support of $b_{n}^{k,h}$ contains at least one complete grid cell inside $\Lambda$, it is called an inner B-spline; otherwise, it is an outer B-spline.

Bivariate B-splines do not naturally satisfy boundary conditions and may lead to ill-conditioned Galerkin matrices. To resolve this, each basis function is multiplied by a smooth weight function

$$\begin{aligned} W(x) = \operatorname {dist}(x, \partial \Lambda ), \quad x \in \Lambda , \end{aligned}$$

vanishing on the boundary (Rvachev function) and outer B-splines are stabilized by combining them with inner ones via correction coefficients $e_{i,j}$, yielding a stable and boundary-compatible basis^5,6^.

##### Definition 2.1

(^6^) For an outer index $j \in J$, let

$$I(j) = j + \{0, \ldots , n\}^2 \subset I$$

be a two-dimensional array of inner indices closest to $j$, assuming $h$ is small enough for such an array to exist. Then,

$$\begin{aligned} e_{i,j} = \prod _{\nu =1}^2 \frac{ \prod _{\begin{array}{c} u=0 \\ u \ne i_\nu - v \end{array}}^{n} (j_\nu - v - u) }{ \prod _{\begin{array}{c} u=0 \\ u \ne i_\nu - v \end{array}}^{n} (i_\nu - v - u) }, \end{aligned}$$

which are the values of the Lagrange polynomials associated with $I(j)$.

As a result, for $i \in I$, the WEB-spline $B_i$ is defined by

$$\begin{aligned} B_i = \frac{W}{W({\textbf {x}}_i)} \left( b_i + \sum _{j \in J} e_{i,j} b_j \right) , \quad b_k = b_n^{k,h}, \end{aligned}$$

where ${\textbf {x}}_i$ is the center of a grid cell fully contained in $\Lambda$ within the support of $b_i$.

#### Galerkin method using WEB-spline basis

Consider the problem defined by the equations (7)-(8) for $k = 1, 2, \ldots , n$. The weak solution satisfies the following variational form:

$$\left( \nabla U^k, \nabla v \right) _{L_2(\Lambda )} + \frac{1}{\lambda } \left( U^k, v \right) _{L_2(\Lambda )} = \left( f_k, v \right) _{L_2(\Lambda )} + \frac{1}{\lambda } \sum _{i=1}^{k-1} \omega _i \left( U^{k-i}, v \right) _{L_2(\Lambda )} + \frac{b_{k-1}}{\lambda } \left( \phi (\textbf{x}), v \right) _{L_2(\Lambda )}.$$

Let the approximate solution $U^k(\textbf{x})$ be

$$\tilde{U}^k(\textbf{x}) = \sum _{i=1}^m c_i^k B_i(\textbf{x}),$$

where $\{B_i(\textbf{x})\}_{i=1}^m$ are the chosen basis functions. Setting the test function $v = B_j(\textbf{x})$ for $j = 1, 2, \ldots , m$, we obtain the discrete system:

$$\begin{aligned} \sum _{i=1}^m c_i^k \left( \nabla B_i, \nabla B_j \right) _{L_2(\Lambda )} + \frac{1}{\lambda } \sum _{i=1}^m c_i^k \left( B_i, B_j \right) _{L_2(\Lambda )}= & \left( f_k, B_j \right) _{L_2(\Lambda )}\\+ & \frac{1}{\lambda } \sum _{i=1}^{k-1} \omega _i \left( \tilde{U}^{k-i}, B_j \right) _{L_2(\Lambda )} + \frac{b_{k-1}}{\lambda } \left( \phi (\textbf{x}), B_j \right) _{L_2(\Lambda )}. \end{aligned}$$

This can be written compactly as the linear system:

$$\sum _{i=1}^m A_{i,j} c_i^k = \textrm{RHS}_j,$$

where

$$\begin{aligned} A_{i,j}&= \left( \nabla B_i, \nabla B_j \right) _{L_2(\Lambda )} + \frac{1}{\lambda } \left( B_i, B_j \right) _{L_2(\Lambda )}, \\ \textrm{RHS}_j&= \left( f_k, B_j \right) _{L_2(\Lambda )} + \frac{1}{\lambda } \sum _{i=1}^{k-1} \omega _i \left( \tilde{U}^{k-i}, B_j \right) _{L_2(\Lambda )} + \frac{b_{k-1}}{\lambda } \left( \phi (\textbf{x}), B_j \right) _{L_2(\Lambda )}. \end{aligned}$$

Thus, at each time step $k$, we solve the following linear of system equations:

$$\underbrace{ \begin{bmatrix} A_{11} & A_{12} & \cdots & A_{1m} \\ A_{21} & A_{22} & \cdots & A_{2m} \\ \vdots & \vdots & \ddots & \vdots \\ A_{m1} & A_{m2} & \cdots & A_{mm} \end{bmatrix} }_{\text {Stiffness matrix}} \begin{bmatrix} c_1^k \\ c_2^k \\ \vdots \\ c_m^k \end{bmatrix} = \begin{bmatrix} \textrm{RHS}_1 \\ \textrm{RHS}_2 \\ \vdots \\ \textrm{RHS}_m \end{bmatrix}.$$

Note that the coefficient matrix remains constant across all iterations, and only the right-hand side vector changes in each time step.

### Numerical example with classical solver

To illustrate the efficiency of the proposed method, we present a numerical example. In this example, we test our computational algorithm for $T=0.1$ and $\alpha =0.6$. For different values of $\alpha$, the results are completely similar. Consider

$$\begin{aligned} & ^CD_t^{0.6} u({\textbf {x}},t) - \Delta u({\textbf {x}},t) = f({\textbf {x}},t), \,\,\,\,\,\,\,\,({\textbf {x}},t) \in {Q_{0.1}},\,\,\,\,\,\,\,\,\,\,\,\,\,\,\,\,\,\,\,\,\,\,\,\,\,\,\,\,\,\,\,\,\,\,\,\,\,\,\,\,\,\,\,\,\\ & u({\textbf {x}},t) = 0, \,\,\,\,\,\,\,\,\,\,\,\,\,\,\,\,\,\,\,\,\,\,\,\,\,\,\,\,\,\,\,\,\,\,\,\,\,\,\,\,\,{\textbf {x}} \in \partial \Lambda ,\,\,t \in (0,0.1),\,\,\,\,\,\,\,\,\,\,\,\,\,\,\,\,\,\,\,\,\,\,\,\,\,\,\,\,\,\,\,\,\,\,\,\,\,\,\,\,\,\,\,\,\,\, \\ & u({\textbf {x}},0) =0,\,\,\,\,\,\,\,\,\,\,\,\,\,\,\,\,\,\,\,\,\,\,\,\,\,\,\,\,\,\,\,\,\,\,\,\,\,\,\,\,\,\,\,\,\,\,\,\,\,\,\,\,\,\,\,\,\,\,\,\,\,\,\,\,\,\,\,\,\,\,\,\,\,\,\,{\textbf {x}} \in \Lambda ,\,\,\,\,\,\,\,\,\,\,\,\,\,\,\,\,\,\,\,\,\,\,\,\,\,\,\,\,\,\,\,\,\,\,\,\,\,\,\,\,\,\,\,\,\,\, \end{aligned}$$

where the exact solution is $u(x,y,t)=100\, t \,x \,y\, (1-x-y)$. For the time discretization, let $t_k = k \tau$ for $k = 0, 1, \ldots , 10$, where $\tau =0.01$. For the spatial discretization, we employ the WEB-spline basis functions of degree one and $h=0.5, 0.25, 0.2$. The weight function used in the WEB-spline finite element method is:

$$W(x,y) =1-\sqrt{x^{2}+y^{2}} -\sqrt{(1-x-y)^{2}+\left( x+y-\sqrt{x^{2}+y^{2}}\right) ^{2}}.$$

For simplicity, we compute the approximate solution $\tilde{U}^{k}(\textbf{x})$ only for $k=1$. Thus, we obtain an approximation to:

$$U^{1} \approx u^{1} = u(x,t_{1}) = u(x,0.01).$$

It is important to emphasize that sparse matrices generally lead to better numerical performance than dense ones. In the WEB-spline finite element method, the stiffness matrix becomes increasingly sparse as the mesh size *h* is refined. However, refining the mesh also increases the number of WEB-spline basis functions and consequently the dimension of the resulting linear system. This directly impacts the number of qubits required when employing quantum linear solvers. For example, when $h = 0.5$, the discretization yields four WEB-spline basis functions; for $h = 0.25$, it yields thirteen basis functions; and for $h = 0.2$, it yields nineteen basis functions. The corresponding stiffness matrices therefore have dimensions:

$$4\times 4,\qquad 13\times 13,\qquad 19\times 19,$$

respectively^5,6,12^. To illustrate the sparsity patterns, we present below symbolic structures of the matrices $A = [a_{i,j}]$, where each “$*$” represents a nonzero entry and each “0” denotes a zero entry. For $h = 0.5$, the $4\times 4$ stiffness matrix has the structure

$$A = [a_{i,j}]_{4\times 4} = \begin{bmatrix} * & * \\ * & * \end{bmatrix}.$$

For $h=0.25$, the $13\times 13$ stiffness matrix has the symbolic nonzero structure

$$A = \left[ \begin{array}{ccccccccccccc} * & * & 0 & 0 & * & * & 0 & 0 & 0 & 0 & 0 & 0 & 0 \\ * & * & * & 0 & * & * & * & 0 & 0 & 0 & 0 & 0 & 0 \\ 0 & * & * & * & 0 & * & * & * & 0 & 0 & 0 & 0 & 0 \\ 0 & 0 & * & * & 0 & 0 & * & * & 0 & 0 & 0 & 0 & 0 \\ * & * & 0 & 0 & * & * & 0 & 0 & * & * & 0 & 0 & 0 \\ * & * & * & 0 & * & * & * & 0 & * & * & * & 0 & 0 \\ 0 & * & * & * & 0 & * & * & * & 0 & * & * & 0 & 0 \\ 0 & 0 & * & * & 0 & 0 & * & * & 0 & 0 & 0 & 0 & 0 \\ 0 & 0 & 0 & 0 & * & * & 0 & 0 & * & * & 0 & * & * \\ 0 & 0 & 0 & 0 & * & * & * & 0 & * & * & * & * & * \\ 0 & 0 & 0 & 0 & 0 & * & * & * & 0 & * & * & 0 & * \\ 0 & 0 & 0 & 0 & 0 & 0 & 0 & 0 & * & * & 0 & * & * \\ 0 & 0 & 0 & 0 & 0 & 0 & 0 & 0 & * & * & * & * & * \end{array} \right]$$

For $h=0.2$, the discretization produces a $19\times 19$ stiffness matrix with the sparsity pattern

$$A = \left[ \begin{array}{ccccccccccccccccccc} * & * & 0 & 0 & 0 & * & * & 0 & 0 & 0 & 0 & 0 & 0 & 0 & 0 & 0 & 0 & 0 & 0 \\ * & * & * & 0 & 0 & * & * & * & 0 & 0 & 0 & 0 & 0 & 0 & 0 & 0 & 0 & 0 & 0 \\ 0 & * & * & * & 0 & 0 & * & * & * & 0 & 0 & 0 & 0 & 0 & 0 & 0 & 0 & 0 & 0 \\ 0 & 0 & * & * & * & 0 & 0 & * & * & * & 0 & 0 & 0 & * & 0 & 0 & 0 & 0 & 0 \\ 0 & 0 & 0 & * & * & 0 & 0 & 0 & * & * & 0 & 0 & 0 & * & 0 & 0 & 0 & 0 & 0 \\ * & * & 0 & 0 & 0 & * & * & 0 & 0 & 0 & * & * & 0 & 0 & 0 & 0 & 0 & 0 & 0 \\ * & * & * & 0 & 0 & * & * & * & 0 & 0 & * & * & * & 0 & 0 & 0 & 0 & 0 & 0 \\ 0 & * & * & * & 0 & 0 & * & * & * & * & 0 & * & * & * & 0 & * & * & 0 & * \\ 0 & 0 & * & * & * & 0 & 0 & * & * & * & 0 & * & * & * & 0 & * & * & 0 & * \\ 0 & 0 & 0 & * & * & 0 & 0 & 0 & * & * & 0 & * & * & * & 0 & * & * & 0 & * \\ 0 & 0 & 0 & 0 & 0 & * & * & 0 & 0 & 0 & * & * & 0 & 0 & * & * & 0 & 0 & 0 \\ 0 & 0 & 0 & 0 & 0 & * & * & * & * & * & * & * & * & 0 & 0 & * & * & 0 & * \\ 0 & 0 & 0 & 0 & 0 & 0 & * & * & * & * & 0 & * & * & * & 0 & * & * & 0 & * \\ 0 & 0 & 0 & * & * & 0 & 0 & * & * & * & 0 & * & * & * & 0 & * & * & 0 & * \\ 0 & 0 & 0 & 0 & 0 & 0 & 0 & 0 & 0 & 0 & * & * & 0 & 0 & * & * & * & * & * \\ 0 & 0 & 0 & 0 & 0 & 0 & 0 & * & * & * & 0 & * & * & * & * & * & * & * & * \\ 0 & 0 & 0 & 0 & 0 & 0 & 0 & * & * & * & 0 & * & * & * & 0 & * & * & * & * \\ 0 & 0 & 0 & 0 & 0 & 0 & 0 & 0 & 0 & 0 & 0 & 0 & 0 & 0 & * & * & * & * & * \\ 0 & 0 & 0 & 0 & 0 & 0 & 0 & * & * & * & 0 & * & * & * & * & * & * & * & * \end{array} \right]$$

**Figure 1:**
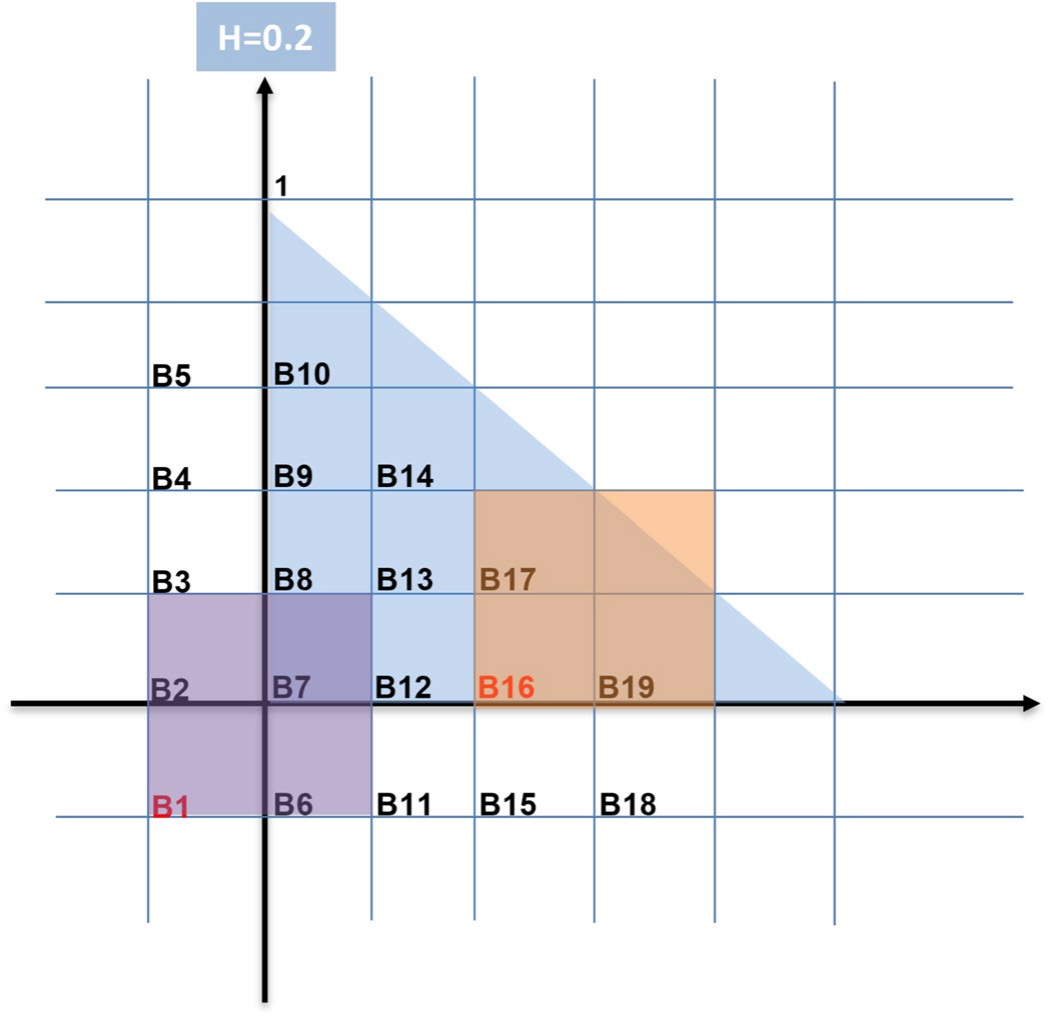
The WEB-spline basis in triangular domain $\Omega = \left\{ {\left( {x,y} \right) \left| {0 \le x \le 1,0 \le y \le 1 - x} \right. } \right\}$ with $h=0.2$.

These examples show that as *h* decreases, the stiffness matrix becomes larger but significantly sparser, a property that is advantageous for both classical and quantum numerical solvers. In the $A = {\left[ {{a_{i,j}}} \right] _{19 \times 19}}$, for example $a_{1,16}=0$ beacuse $\text {support}B_{1}\cap \text {support}B_{16}=\emptyset$ (Ses Figure 1).

Table 1 summarizes the residual norm $\Vert A x^{*}-b\Vert$ for the exact classical solution $x^{*}$ together with the $L_{2}$-norm error of the WEB-spline finite element method (WEBFEM) using a classical linear solver for $m=4, 13, 19$ and $n=10$. As the mesh is refined (i.e., as *h* decreases), the dimension of the resulting linear system increases. Furthermore, the condition number $\kappa (A)$ increases only moderately with refinement, indicating that the matrices remain well-conditioned and suitable for iterative and quantum solvers. The results clearly demonstrate that the approximate WEBFEM solution yields an accurate representation of the desired solution, with the accuracy improving as the mesh is refined.

**Table 1:** Performance summary for different mesh sizes.

*h*	Size	$\Vert A x^{*}-b\Vert$	$\kappa (A)$	$L_{2}$-norm WEBFEM
0.5	$4\times 4$	$5.551\times 10^{-17}$	14.1634	$4.4898\times 10^{-3}$
0.25	$13\times 13$	$7.008\times 10^{-17}$	22.6278	$1.3520\times 10^{-3}$
0.2	$19\times 19$	$1.025\times 10^{-16}$	24.8521	$7.8854\times 10^{-4}$

**Figure 2:**
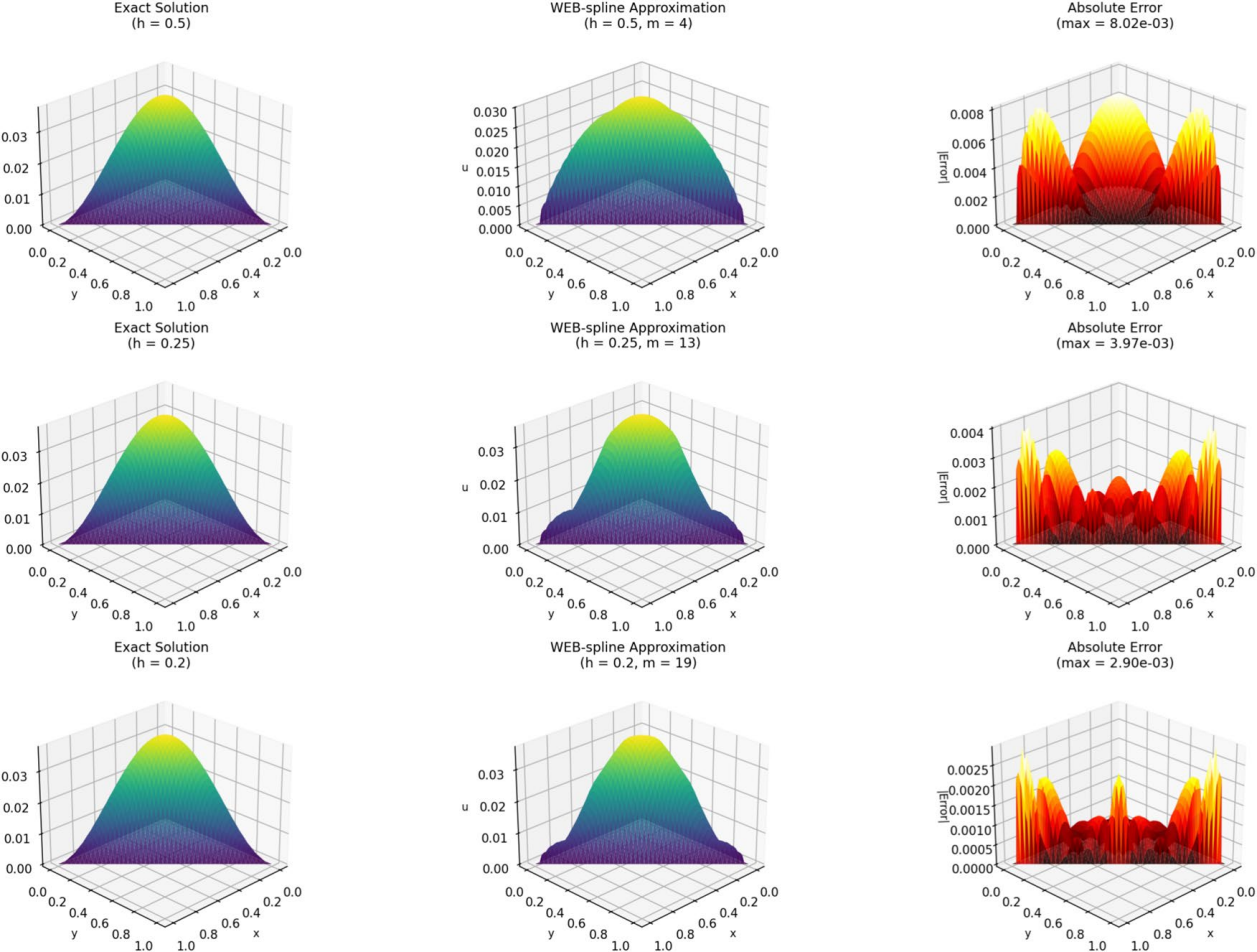
3D solution comparision: Exact vs WEBFEM for $t=0.01$.

**Figure 3:**
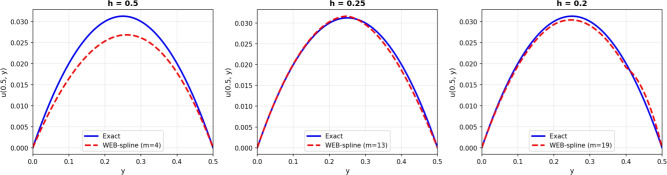
Solution profile at the cross-section $x = 0.5$ for $t = 0.01$.

**Figure 4:**
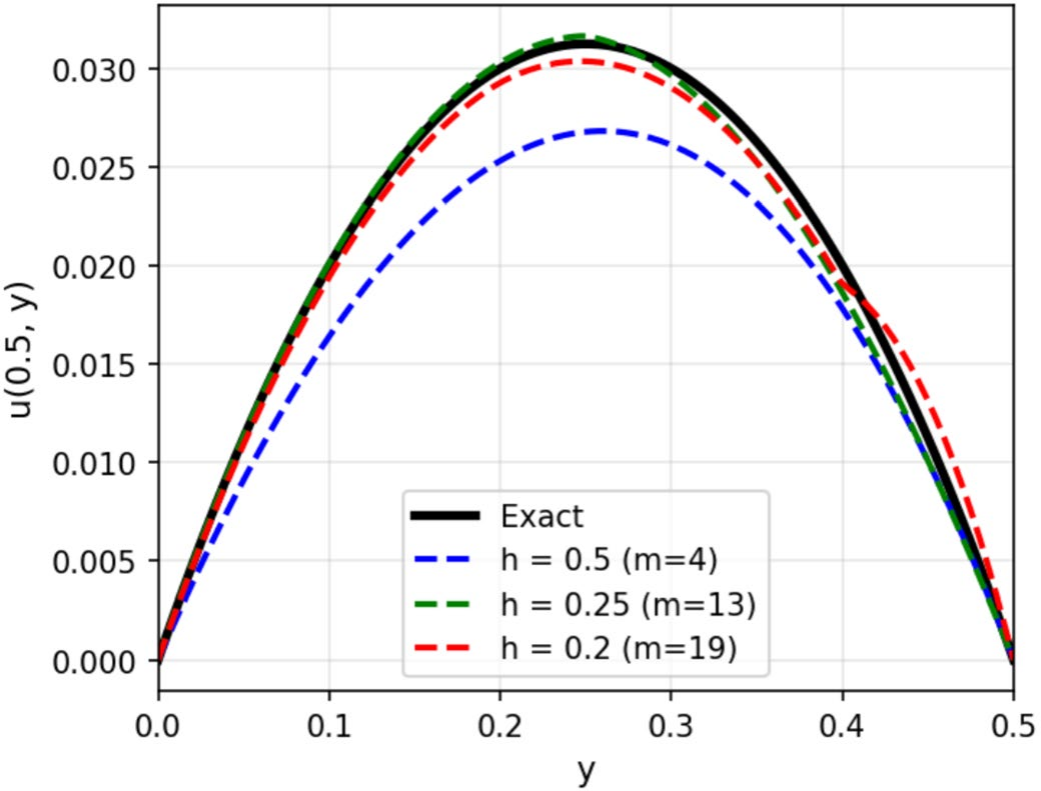
All solutions profile at the cross-section $x = 0.5$ for $t = 0.01$.

Figure 2 presents the three-dimensional solution comparison between the exact solution and the numerical approximation obtained using the WEB-spline finite element method at time $t = 0.01$. The WEBFEM surface closely follows the exact solution, capturing both the shape and amplitude of the solution with high accuracy. The agreement between the two surfaces demonstrates the ability of the WEB-spline basis to approximate smooth transient solutions effectively, even on relatively coarse meshes. To further illustrate the accuracy of the method, Figures 3 and 4 shows the solution profile at the cross-section $x = 0.5$ for $t = 0.01$. The numerical profile aligns very well with the exact one, with only minor deviations near regions where the gradient is steepest. This close agreement confirms the convergence behavior observed in the global error analysis and provides a clear visual validation of the reliability of the WEBFEM approximation.

## Variational quantum algorithm for solving linear system of equations

The objective is to solve the linear system of equations:

$$A x = b, \quad A \in \mathbb {C}^{N \times N}, \quad b \in \mathbb {C}^N,$$

where $A$ is Hermitian, or can be embedded into a Hermitian matrix if necessary. In the quantum framework, both the right-hand side vector $b$ and the solution vector $x$ are represented as normalized quantum states:

$$|b\rangle = \frac{b}{\Vert b\Vert }, \quad |x\rangle = \frac{x}{\Vert x\Vert }.$$

The task then becomes the preparation of a quantum state $|x\rangle$ that approximates the normalized solution. This formulation naturally lends itself to near-term quantum devices, where the solution is encoded in the amplitudes of qubits.

### Ansatz for the Solution

Since the inverse operation $A^{-1}$ is non-unitary and cannot be directly implemented on quantum hardware, the variational approach approximates the solution by preparing a parameterized quantum state. This is achieved through an ansatz circuit:

13 $$\begin{aligned} |x(\theta )\rangle = U(\theta )|0\rangle , \end{aligned}$$

where $U(\theta )$ is a unitary operator constructed from parameterized gates, such as rotations and entangling operations. The specific choice of ansatz depends on the problem at hand; hardware-efficient circuits or problem-inspired ansatze can be employed to enhance performance. The expressiveness of the ansatz significantly influences the quality of the solution^22^.

In order to direct the optimization, we define a cost function that becomes zero if and only if $|x(\theta )\rangle$ coincides (up to normalization) with the true solution. A suitable and commonly used choice is the residual norm:

$$C(\theta ) = \Vert A|x(\theta )\rangle - |b\rangle \Vert ^2.$$

Expanding this expression yields measurable expectation values:

$$C(\theta ) = \langle x(\theta )| A^\dagger A |x(\theta )\rangle - 2 \, \Re \big (\langle x(\theta )| A^\dagger |b\rangle \big ) + 1.$$

Each term corresponds to an observable that can be estimated on a quantum computer. In practice, this involves preparing the state $|x(\theta )\rangle$ and measuring with respect to operators derived from $A$ and $|b\rangle$^22^.

The algorithm operates within a hybrid quantum-classical loop. At each iteration, the quantum device prepares the trial state $|x(\theta )\rangle$ and estimates the cost function (and optionally its gradient). This information is then passed to a classical optimizer, which updates the parameters $\theta$. The cycle repeats until the cost converges below a specified tolerance. The outcome is a set of parameters $\theta ^*$ for which the ansatz state $|x(\theta ^*)\rangle$ approximates the desired solution $|x\rangle$. The variational nature of the algorithm allows it to function effectively on noisy, near-term hardware^22^.

In the following, the algorithm of VQLS for solving a linear system of equations is given:

**Algorithm 1:**
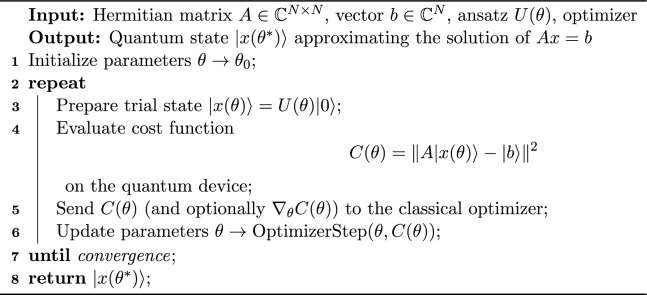
Variational Quantum Linear Solver (VQLS)

### Parameterized Quantum Circuit $U(\theta )$

In VQLS^22^, the trial solution state is expressed as (13) where $U(\theta )$ is a parameterized unitary operator (commonly referred to as the ansatz) and $|0\rangle$ denotes the initial reference state of all qubits. The vector of parameters $\theta = (\theta _1, \theta _2, \ldots , \theta _m)$ controls the action of $U(\theta )$, typically corresponding to rotation angles of quantum gates.

The unitary $U(\theta )$ is constructed as a sequence of layers combining parameterized single-qubit rotations and entangling gates. A generic layered structure is given by:

$$U(\theta ) = \prod _{l=1}^L \left( \prod _{i=1}^n R_y(\theta _{i,l}) R_z(\theta _{i,l}) \right) \cdot \text {Entanglers},$$

where $n$ is the number of qubits, $L$ is the number of layers, and *Entanglers* denote multi-qubit gates such as controlled-NOT (CNOT) or controlled-Z operations^23^. This layered design enables $U(\theta )$ to explore a large subspace of the Hilbert space while keeping the circuit depth relatively shallow, which is important for near-term quantum devices.

The choice of ansatz strongly affects algorithm performance. If $U(\theta )$ is too simple, it may fail to represent the true solution state $|x\rangle$; if it is overly complex, optimization becomes difficult and noise effects are amplified. Thus, $U(\theta )$ serves a role analogous to a trial function in classical variational methods, balancing expressiveness and trainability. For example, for a two-qubit system, one may define

$$U(\theta ) = \Big ( R_y(\theta _1) \otimes R_y(\theta _2) \Big ) \cdot \text {CNOT}_{1,2} \cdot \Big ( R_y(\theta _3) \otimes R_y(\theta _4) \Big ),$$

which prepares a variational state depending on four parameters $\{\theta _1,\theta _2,\theta _3,\theta _4\}$. These parameters are optimized iteratively within the hybrid quantum-classical loop to minimize the cost function and approximate the solution to $Ax = b$.

### Numerical example with VQLS

Consider the linear systems obtained from the WEB-spline finite element discretization. For a system of dimension *N*, the number of qubits required to encode the solution vector is given by $n_q = \lceil \log _2 N \rceil$. Table 2 summarizes the corresponding qubit requirements for the WEB-spline discretizations examined in this study.

**Table 2:** Qubit requirements for VQLS based on WEB-spline system size.

*h*	*N*	Qubits required	Hilbert space dim.
0.5	4	2	4
0.25	13	4	16
0.2	19	5	32

In the following, we give the step by step of the algorithm:

**Step 1: Normalization and state preparation:** The right-hand side vector is encoded into a normalized quantum state:

$$|b\rangle = \frac{\textbf{b}}{\Vert \textbf{b}\Vert }, \qquad \Vert \textbf{b}\Vert = \sqrt{\sum _{i=1}^{N} b_i^{2}} .$$

To prepare this state on a quantum computer, we introduce a unitary operator $U_b$ satisfying

$$U_b |0\rangle = |b\rangle ,$$

where the number of qubits required for the encoding is $n_q = \lceil \log _2 N \rceil$, ensuring that the computational basis provides at least *N* amplitudes for representing the vector.

**Step 2: Ansatz for the solution state:** The unknown solution vector is represented variationally as:

$$|x(\theta )\rangle = U(\theta )\,|0\rangle ,$$

where $U(\theta )$ denotes a parameterized ansatz circuit acting on $n_q = \lceil \log _2 N \rceil$ qubits. Typical ansatz constructions employ layered structures composed of single-qubit rotations (e.g., $R_y(\theta )$ or $R_z(\theta )$ gates) combined with entangling operations such as CNOT gates^22,24^. The set of variational parameters $\theta$ is optimized so that the trial state $|x(\theta )\rangle$ approximates the normalized solution vector $\textbf{c}/\Vert \textbf{c}\Vert$ as closely as possible.

**Step 3: Cost function:** The variational cost is defined as:

$$C(\theta ) = \big \Vert A|x(\theta )\rangle - |b\rangle \big \Vert ^2,$$

which expands to

$$C(\theta ) = \langle x(\theta )|A^\dagger A|x(\theta )\rangle - 2\,\Re \,\big (\langle x(\theta )|A^\dagger |b\rangle \big ) + 1.$$

Here the term $\langle x|A^\dagger A|x\rangle$ is estimated by decomposing $A^\dagger A$ into Pauli strings and measuring expectation values. and the overlap $\langle x|A^\dagger |b\rangle$ can be obtained via the Hadamard test^25^.

**Step 4: Variational optimization:** A classical optimizer (e.g., COBYLA or BFGS^26^) iteratively updates the parameter vector $\theta$ by repeatedly preparing the trial state $|x(\theta )\rangle$ on the quantum device, evaluating the cost function $C(\theta )$ through quantum measurements and adjusting the parameters according to $\theta \mapsto \theta '$ to decrease the objective value. This hybrid quantum–classical loop is executed until convergence, typically characterized by achieving a parameter set $\theta ^*$ for which $C(\theta ^*) \approx 0$.

**Step 5: Extracting the solution vector:** At convergence, the state encodes the normalized solution:

$$|x(\theta ^*)\rangle = \frac{\textbf{c}}{\Vert \textbf{c}\Vert }.$$

The classical solution $\textbf{c}$ can be reconstructed as $\textbf{c} = \gamma \, x_{\text {measured}}$ where $x_{\text {measured}}$ is obtained via quantum state tomography and $\gamma$ is a scaling factor determined from $\Vert \textbf{b}\Vert$ and the conditioning of *A*.

Now, we benchmarked VQLS with different ansatz configurations. Table 3 summarizes the performance in terms of the residual norm $\Vert A x_{\textrm{VQLS}} - b\Vert$ compared to the exact classical solution $x^{*}$ for systems of sizes $m = 4, 13, 19$ with $n = 10$. Classical computations were carried out on a desktop computer equipped with a Pentium Dual-Core CPU E5700 running at 3.00 GHz, while the quantum implementation of the method was simulated using the IBM Quantum simulator via Qiskit. In addition to accuracy, Table 3 provides detailed information on the optimization effort, including the number of iterations required to reach convergence and the total run time for each configuration. For the VQLS results reported in Table 3, the measured runtimes, which reach approximately 320s for the largest system, represent the complete hybrid quantum-classical optimization process. This includes all optimizer iterations, which can exceed 7000 steps for the larger problem instances, the repeated execution of quantum circuits required to evaluate the cost function at every iteration and the associated classical gradient computation and parameter updates. Consequently, these values reflect the full end-to-end wall-clock time required to obtain the final solution. These metrics highlight the interplay between ansatz expressivity and computational cost.

**Table 3:** Summary of VQLS performance across WEBFEM systems.

*h*	Size	Qubits	Layers	Params	$\Vert A x_{VQLS} - b\Vert$	Iters	Time (s)
0.5	$4\times 4$	2	1	2	$8.85\times 10^{-3}$	255	2.06
0.5	$4\times 4$	2	2	4	$5.64\times 10^{-10}$	372	4.23
0.25	$13\times 13$	4	1	4	$4.99\times 10^{-2}$	431	4.43
0.25	$13\times 13$	4	2	8	$1.85\times 10^{-2}$	1298	21.81
0.25	$13\times 13$	4	3	12	$5.18\times 10^{-8}$	7084	171.07
0.2	$19\times 19$	5	1	5	$1.04\times 10^{-1}$	528	5.27
0.2	$19\times 19$	5	2	10	$5.15\times 10^{-2}$	891	29.84
0.2	$19\times 19$	5	3	15	$2.21\times 10^{-2}$	6588	186.82
0.2	$19\times 19$	5	4	20	$1.76\times 10^{-7}$	4254	320.705

Table 4 provides a comprehensive comparison across all mesh sizes, showing the best-performing ansatz configuration for each case.

**Table 4:** Best-performing VQLS ansatz configurations for each WEBFEM system.

*h*	Size	Best Ansatz	$\Vert A x_{VQLS} - b\Vert$	Time (s)
0.5	$4\times 4$	2q, 2L	$5.64\times 10^{-10}$	4.23
0.25	$13\times 13$	4q, 3L	$4.26\times 10^{-8}$	171.07
0.2	$19\times 19$	5q, 4L	$1.76\times 10^{-7}$	320.705

**Figure 5:**
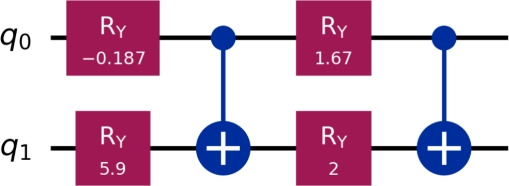
Best-performing VQLS ansatz configurations for $h=0.5$, i.e., 2*q*, 2*L*.

**Figure 6:**
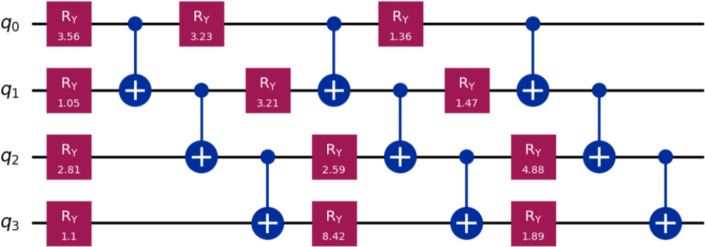
Best-performing VQLS ansatz configurations for $h=0.25$, i.e., 4*q*, 3*L*.

**Figure 7:**
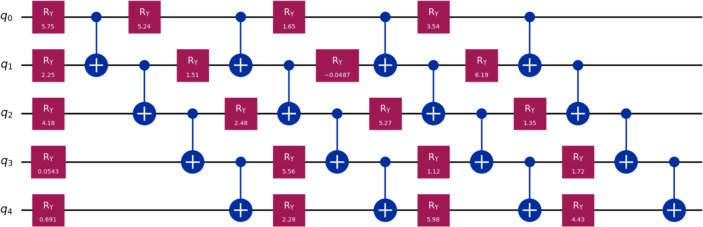
Best-performing VQLS ansatz configurations for $h=0.2$, i.e., 5*q*, 4*L*.

The circuits in Figures 5, 6, 7 illustrate how the best-performing VQLS ansatze scale with system size. For $h=0.5$, a compact two-qubit, two-layer circuit with four parameters achieves near-exact accuracy ($5.64\times 10^{-10}$). The $h=0.25$ system requires a more expressive four-qubit, three-layer ansatz with 12 parameters, reducing the residual to $4.26\times 10^{-8}$. For the largest case ($h=0.2$), a five-qubit, four-layer circuit with 20 parameters is needed to capture the solution structure, achieving $1.76\times 10^{-7}$. This progression highlights the expected scaling of VQLS: larger WEBFEM systems demand deeper and more expressive parameterized circuits.

**Figure 8:**
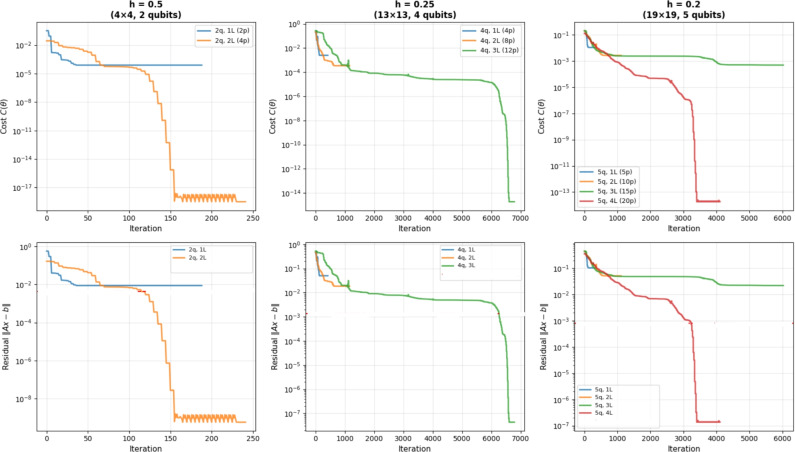
VQLS convergence for WEB-spline FEM systems.

The convergence plot in Figure 8 shows that VQLS systematically reduces the residual error associated with the WEBFEM discretization, demonstrating stable optimization behavior across increasing circuit depth.

**Figure 9:**
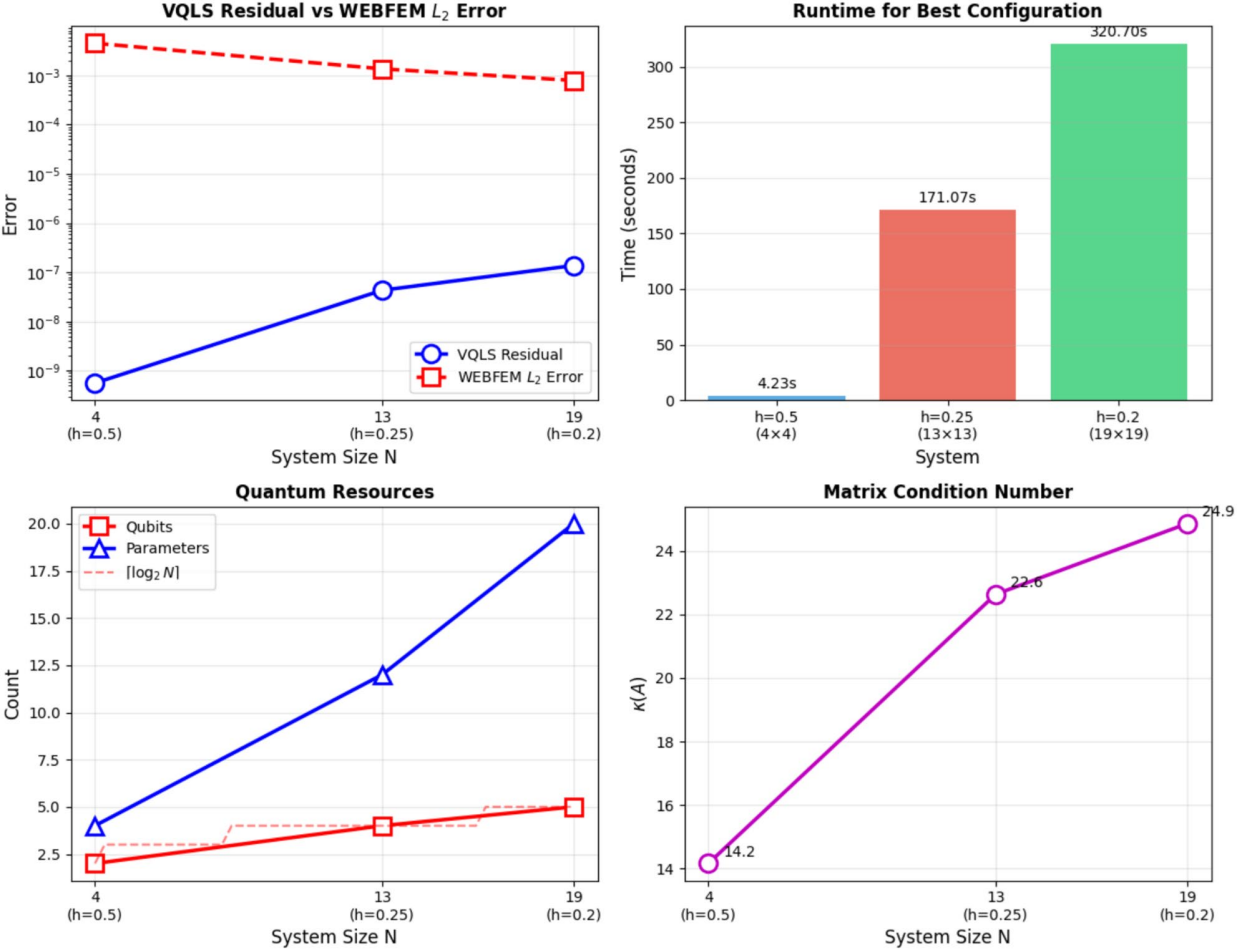
VQLS scaling analysis.

The scaling analysis in Figure 9 highlights several key trends. The VQLS residual closely tracks the WEBFEM $L^{2}$ error, indicating that reductions in the variational loss correspond to meaningful improvements in physical accuracy. Runtime increases with problem size but remains moderate, showing that the hybrid optimization loop scales efficiently for the tested systems. Quantum resource requirements grow gradually, with qubit and parameter counts increasing in line with system dimension while still remaining compact relative to problem size. The condition numbers of the WEBFEM matrices rise with *N*, reflecting increasing numerical difficulty and providing useful context for VQLS convergence. Overall, the results demonstrate that VQLS scales smoothly with system size and effectively captures the structure of WEB-spline discretizations.

## HHL quantum algorithm for solving linear system of equations

Cosider the following linear system of equations:

$$A x = b, \quad A \in \mathbb {C}^{N \times N}, \quad x, b \in \mathbb {C}^{N}.$$

Solving the linear system $A x= b$ using classical methods, such as Gaussian elimination or LU decomposition, generally requires $\mathcal {O}(N^3)$ operations for a dense matrix, making it computationally expensive for large-scale problems. HHL quantum algorithm^8^ provides a potential exponential speedup by encoding the solution vector *x* into a quantum state $\vert {x} \rangle$ and performing linear algebra operations in the quantum domain. For a sparse, Hermitian, and well-conditioned matrix *A*, the HHL algorithm can prepare a quantum state proportional to the solution in time scaling as $\mathcal {O}(\log N\,\kappa ^2 s^2/\epsilon )$, where $N$ is the system size, $\kappa$ is the condition number of $A$, $\epsilon$ is the desired precision, and $s$ denotes the sparsity (number of non-zero entries per row/column). This represents an exponential improvement in *N* compared to classical methods, though the speedup depends on efficient state preparation, Hamiltonian simulation, and low condition numbers. The HHL algorithm has applications in quantum machine learning, differential equations, and optimization, making it a cornerstone example of quantum advantage in linear algebra.

### Mathematical foundations

Let $A$ be a Hermitian matrix with the spectral decomposition

$$\begin{aligned} A = \sum _{j=0}^{m-1} \lambda _j \vert {u_j} \rangle \langle {u_j}\vert , \end{aligned}$$

where $\lambda _j$ are the eigenvalues and $\{\vert {u_j} \rangle \}$ the corresponding orthonormal eigenvectors. Since $A$ is diagonalizable in this eigenbasis, its inverse can be expressed as

$$\begin{aligned} A^{-1} = \sum _{j=0}^{m-1} \lambda _j^{-1} \vert {u_j} \rangle \langle {u_j}\vert . \end{aligned}$$

If the vector $\vert {b} \rangle$ is decomposed in the same basis as

$$\begin{aligned} \vert {b} \rangle = \sum _{j=0}^{m-1} \beta _j \vert {u_j} \rangle , \end{aligned}$$

then the solution to the linear system is

$$\begin{aligned} \vert {x} \rangle = A^{-1} \vert {b} \rangle = \sum _{j=0}^{m-1} \frac{\beta _j}{\lambda _j} \vert {u_j} \rangle . \end{aligned}$$

This solution can be encoded approximately as a quantum state using the HHL algorithm.

### Structure of the HHL algorithm

The structure of HHL algorithm can be divided into four main stages: (i) *state preparation*, where the right-hand side vector *b* is encoded into a quantum state $\vert {b} \rangle$; (ii) *quantum phase estimation (QPE)*, which decomposes $\vert {b} \rangle$ in the eigenbasis of *A* and stores the corresponding eigenvalues in an ancillary register; (iii) *controlled rotation*, where a rotation proportional to the reciprocal of each eigenvalue is applied to an ancilla qubit, effectively performing the inversion of *A*; and (iv) *uncomputation and post-selection*, which disentangles the ancilla and eigenvalue registers, leaving the system in a state proportional to the solution $\vert {x} \rangle$. This modular structure allows each stage to be implemented efficiently on a quantum computer for sparse, well-conditioned matrices.

In the following, the algorithm of HHL for solving a linear system of equations is given:

**Algorithm 2:**
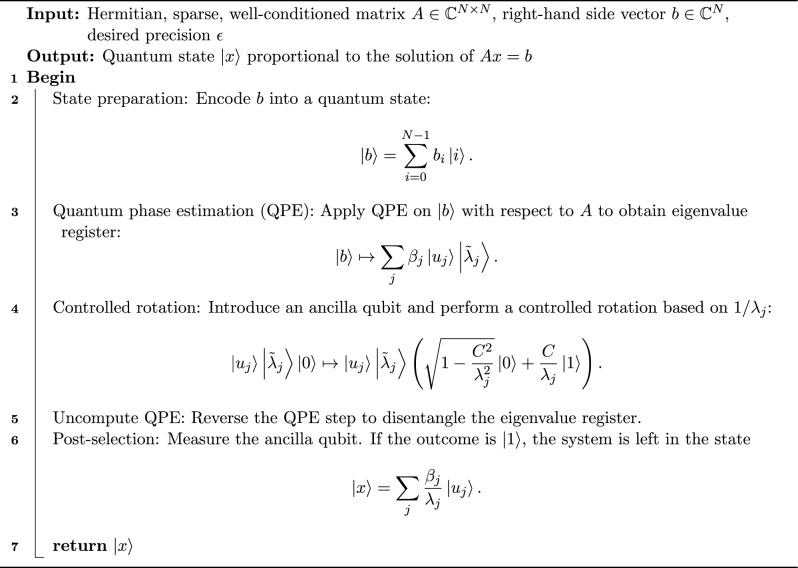
HHL quantum algorithm for solving $A x =b$

### Numerical example with HHL

Consider the linear systems obtained from the WEB-spline finite element discretization. In the following, we give the step by step of the algorithm:

**Step 1: State Preparation:** Encode the right-hand side *b* into quantum state $|b\rangle$ using $n_b = \lceil \log _2 N\rceil$ qubits.

**Step 2: Hamiltonian simulation:** Implement the unitary evolution $e^{iAt}$ for a suitable time *t*, which encodes the action of *A* into a quantum operation.

**Step 3: QPE:** Apply QPE to decompose $\vert {b} \rangle$ in the eigenbasis of *A*, storing approximate eigenvalues $\tilde{\lambda }_j$ in an ancillary register:

$$\vert {b} \rangle \mapsto \sum _{j=0}^{3} \beta _j \vert {u_j} \rangle \vert {\tilde{\lambda }_j} \rangle ,$$

where $\vert {u_j} \rangle$ are eigenvectors of *A* and $\beta _j = \langle {u_j|b}\vert {u_j|b} \rangle$. In other words, extract eigenvalues of *A* using $n_{\text {clock}}$ auxiliary qubits, achieving precision $2^{-n_{\text {clock}}}$.

**Step 4: Controlled rotation:** Perform a rotation on an ancilla qubit conditioned on the eigenvalue register, effectively applying $A^{-1}$:

$$\vert {u_j} \rangle \vert {\tilde{\lambda }_j} \rangle \vert {0} \rangle \mapsto \vert {u_j} \rangle \vert {\tilde{\lambda }_j} \rangle \Big (\sqrt{1-\frac{C^2}{\lambda _j^2}}\vert {0} \rangle + \frac{C}{\lambda _j}\vert {1} \rangle \Big ),$$

where *C* is a normalization constant.

**Step 5: Uncompute QPE:** Reverse the QPE step to disentangle the eigenvalue register.

**Step 6: Post-selection:** Measure the ancilla qubit. If the outcome is $\vert {1} \rangle$, the system qubits are left in the state

$$\vert {x} \rangle = \sum _{j=0}^{m-1} \frac{\beta _j}{\lambda _j} \vert {u_j} \rangle ,$$

which encodes the solution vector.

**Step 7: Measurement / Extraction:** Extract the solution either by measuring the system qubits directly or performing quantum state tomography to reconstruct the amplitudes.

Again, classical computations were performed on a desktop machine equipped with a Pentium(R) Dual-Core CPU E5700 at 3.00 GHz. The quantum version of the method was simulated using the IBM Quantum simulator via Qiskit, providing a platform to verify both the correctness and efficiency of the algorithm prior to execution on actual quantum hardware. Consequently, the HHL algorithm enables the solution of the linear system derived from the proposed method. The computed solution is given in Table 5. The HHL timings listed in Table 5, on the order of $3\times 10^{-5}$ to $8\times 10^{-5}$s, correspond solely to the execution time of a single compiled quantum circuit evaluated using the statevector simulator. These measurements do not include the overhead associated with state preparation, quantum state tomography for extracting the classical solution, or circuit compilation. They therefore represent an idealized circuit execution time rather than the total algorithmic runtime.

**Table 5:** Results of the HHL algorithm for solving the linear system.

*h*	Size	$n_b$	$n_{\text {clock}}$	Qubits	$\Vert A x_{HHL} - b\Vert$	Time (s)
0.5	$4\times 4$	2	4	7	$3.07\times 10^{-3}$	0.000047
0.5	$4\times 4$	2	6	9	$9.56\times 10^{-4}$	0.000031
0.5	$4\times 4$	2	8	11	$2.95\times 10^{-4}$	0.000031
0.5	$4\times 4$	2	10	13	$4.66\times 10^{-5}$	0.000029
0.25	$13\times 13$	4	4	9	$3.06\times 10^{-2}$	0.000057
0.25	$13\times 13$	4	6	11	$1.27\times 10^{-2}$	0.000079
0.25	$13\times 13$	4	8	13	$2.41\times 10^{-3}$	0.000053
0.25	$13\times 13$	4	10	15	$6.46\times 10^{-4}$	0.000046
0.2	$19\times 19$	5	4	10	$5.36\times 10^{-2}$	0.000061
0.2	$19\times 19$	5	6	12	$9.37\times 10^{-3}$	0.000066
0.2	$19\times 19$	5	8	14	$4.29\times 10^{-3}$	0.000055
0.2	$19\times 19$	5	10	16	$9.02\times 10^{-4}$	0.000052

Table 6 summarizes the quantum gate counts required by the HHL algorithm for the WEBFEM linear systems. The gate complexity increases with decreasing mesh size, reflecting the higher dimensionality and deeper QPE circuits required for larger systems.

**Table 6:** HHL circuit gate counts for WEBFEM systems.

*h*	Size	$n_b$	$n_{\text {clk}}$	Qubits	H	CX	CP	CRY	SWAP	Total	Depth
0.5	$4\times 4$	2	8	11	32	15	88	8	8	153	84
0.25	$13\times 13$	4	8	13	32	17	120	8	8	189	104
0.2	$19\times 19$	5	8	14	32	18	136	8	8	207	114

**Figure 10:**
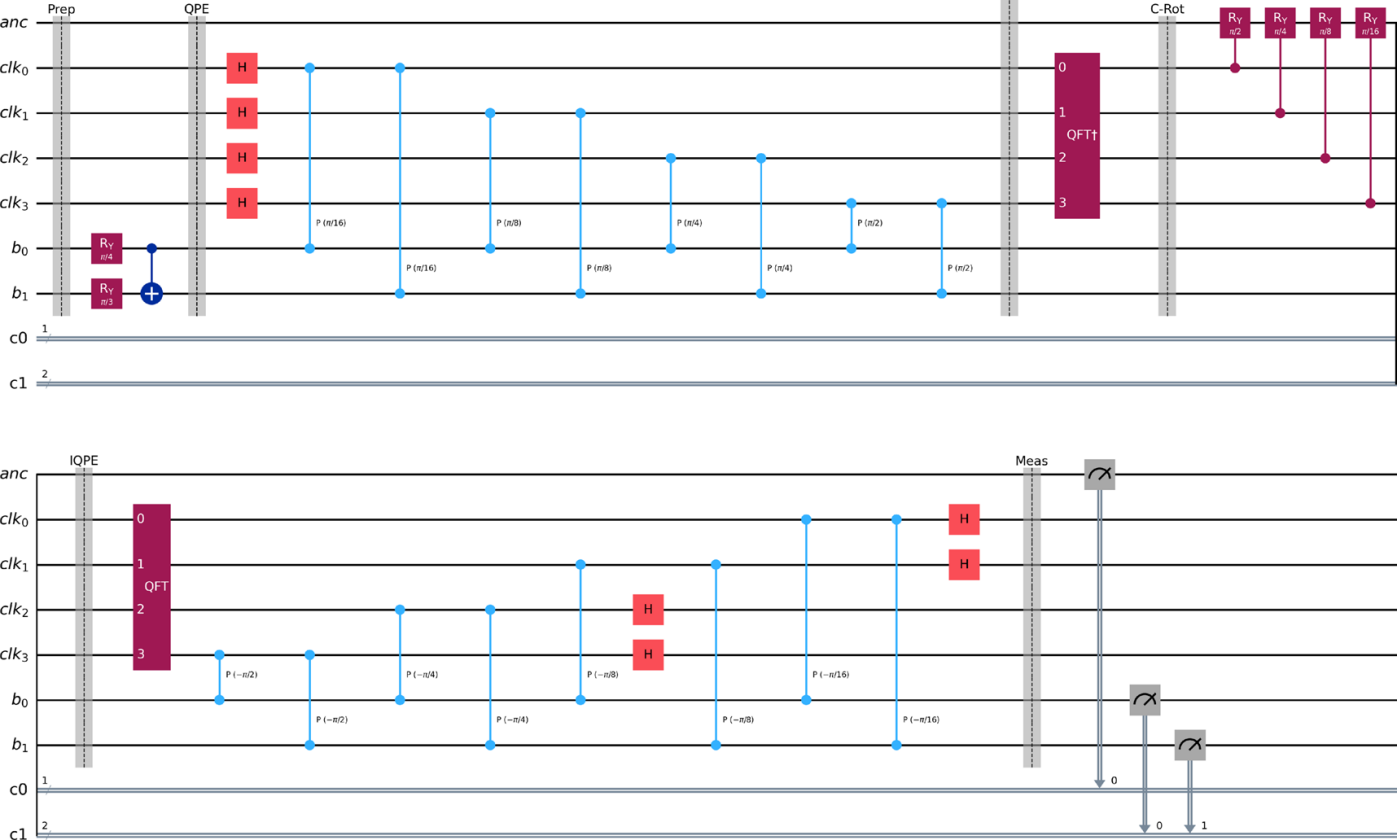
The circuit that implements the HHL quantum algorithm $h=0.5$, $n_b=2$ and $n_{\text {clk}}=4$.

The quantum circuit implementing the HHL algorithm for the case $h = 0.5$ and the $4\times 4$ WEBFEM system is shown in Figure 10. For this configuration, the HHL circuit uses $n_b = 2$ eigenvalue-register qubits and $n_{\text {clock}} = 4$ phase-estimation clock qubits, resulting in a total of 7 qubits when including the state-register and ancilla qubits required for the controlled rotation. The quantum solution obtained from this circuit closely matches the corresponding classical solution, with only a small deviation attributable to the finite precision of the QPE encoding.

**Figure 11:**
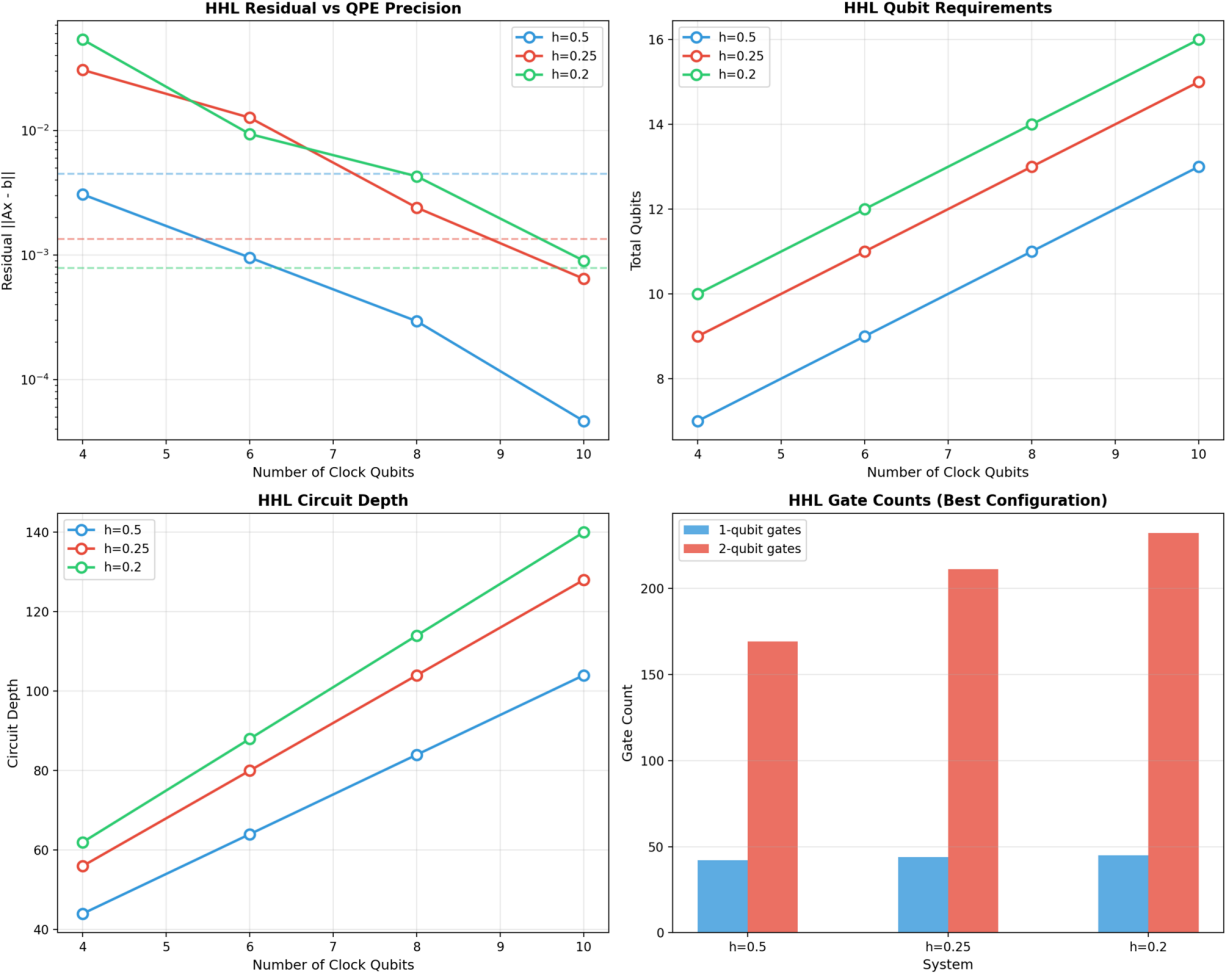
HHL algorithm analysis.

Figure 11 summarizes the scaling behavior of the HHL algorithm for WEBFEM systems. Increasing the number of QPE clock qubits consistently improves the solution residual, with the smallest system showing the fastest accuracy gain. Qubit counts grow linearly with QPE precision, reflecting the combined requirements of the eigenvalue, state, and ancilla registers. Circuit depth likewise increases nearly linearly and is systematically larger for finer FEM discretizations. The gate-count analysis confirms that two-qubit controlled operations dominate the computational cost, with both one-qubit and two-qubit gate counts rising significantly as the system size increases. Collectively, these trends highlight the steep resource demands of HHL and the practical challenges of applying QPE-based quantum linear solvers to larger PDE discretizations.

## Quantum annealing for solving linear system of equations

Linear systems of equations lie at the heart of countless applications in science, engineering, optimization, and data analysis. Classical algorithms such as Gaussian elimination, LU decomposition, or iterative Krylov subspace methods have been widely used to solve these problems, but they often become computationally expensive when the system size grows, particularly for large, sparse, or ill-conditioned matrices^28,29^. With the rise of quantum computing, researchers are exploring novel paradigms that can potentially outperform classical solvers by leveraging quantum parallelism and non-classical dynamics. Among these paradigms, quantum annealing provides a physically motivated framework for finding approximate solutions to optimization problems, including the reformulation of linear systems^14,30^.

Quantum annealing approaches the problem by mapping the solution of $A x = b$ into an equivalent optimization task, typically through the minimization of a quadratic cost function $\Vert Ax - b\Vert ^2$. This formulation can then be expressed as a QUBO problem, which quantum annealers such as D-Wave machines are designed to address^31,32^. By encoding the system into the energy landscape of a quantum Hamiltonian, the annealer evolves toward a ground state that approximates the solution vector. While still approximate and limited by hardware constraints, quantum annealing opens a promising pathway for solving large-scale linear systems efficiently, particularly in contexts where classical iterative methods struggle due to ill-conditioning or high dimensionality^33,34^.

### Mathematical foundations

Minimize a function *f*(*x*) where $x \in \{0,1\}^n$. Quantum annealing solves this by mapping it to an Ising model or QUBO form^35,36^. The Ising model is a fundamental mathematical model originally developed in statistical physics to describe ferromagnetism. In quantum annealing, it is used to formulate and solve combinatorial optimization problems by minimizing an energy function, called the Hamiltonian. The standard form of the Ising model Hamiltonian is:

$$\begin{aligned} H(s) = \sum _{i} h_i s_i + \sum _{i < j} J_{ij} s_i s_j , \end{aligned}$$

where $s_i \in \{-1, +1\}$ are spin variables, $h_i \in \mathbb {R}$ are local biases or external magnetic fields, $J_{ij} \in \mathbb {R}$ are coupling coefficients between spins $s_i$ and $s_j$, and *H*(*s*) is the total energy (cost) of a spin configuration *s*. From a physical point of view, $h_i s_i$ represents the energy contribution from the alignment of spin $s_i$ with an external field, and $J_{ij} s_i s_j$ corresponds to the interaction energy between spins^10,35^. If $J_{ij} > 0$, spins prefer to align (ferromagnetic interaction) and if $J_{ij} < 0$, spins prefer to anti-align (antiferromagnetic interaction). From a computational viewpoint, the Ising model is treated as an optimization problem:

$$\begin{aligned} \text {Minimize } H(s) = \sum _{i} h_i s_i + \sum _{i < j} J_{ij} s_i s_j, \quad \text {where } s_i \in \{-1, +1\}. \end{aligned}$$

QUBO refers to optimization problems of the form:

$$\begin{aligned} \min _{x \in \{0,1\}^n} x^T Q x , \end{aligned}$$

where $x = (x_1, x_2, \dots , x_n)^T$ is a vector of binary variables with $x_i \in \{0,1\}$, $Q \in \mathbb {R}^{n \times n}$ is a symmetric matrix of real-valued coefficients, and $x^T Q x$ is a quadratic form representing the cost function to minimize. The matrix *Q* defines two types of terms, linear terms (diagonal) $Q_{ii} \cdot x_i$ and quadratic terms (off-diagonal) $Q_{ij} \cdot x_i x_j$, for $i \ne j$. Hence, the full objective function can be written as:

$$\begin{aligned} x^T Q x = \sum _{i=1}^{n} Q_{ii} x_i + \sum _{i<j} Q_{ij} x_i x_j . \end{aligned}$$

This formulation is commonly used in combinatorial optimization and is especially well-suited for implementation on quantum annealers^10^.

### Structure of the quantum annealing process

Solving a linear system of equations using quantum annealing requires reformulating the problem into a QUBO structure suitable for implementation on quantum annealers. Consider the linear system $A x = b,$ which can be recast as an optimization problem by minimizing the squared residual:

14 $$\begin{aligned} x_{\text {sol}} = \arg \min _x \Vert Ax-b\Vert ^2 = \arg \min _x (Ax-b)^\dagger (Ax-b). \end{aligned}$$

This formulation is quadratic in the variables $x_i$, aligning naturally with the QUBO framework. However, quantum hardware operates on binary variables, and therefore a binary representation of the real-valued solution vector *x* is required. Each component $x_i$ is encoded using *R* binary variables $q_i^r \in \{0,1\}$ through a scaled and shifted binary encoding:

15 $$\begin{aligned} x_i = c_i \sum _{r=1}^R q_i^r 2^{-r} - d_i, \end{aligned}$$

where $c_i$ is a scaling factor controlling resolution, $d_i$ is a shift term enabling representation of signed numbers, and $q_i^r$ is the *r*-th binary variable representing the *i*-th component. By choosing $d_i > 0$ and $c_i > d_i/2$, both positive and negative values can be represented while minimizing qubit usage, which is crucial for NISQ-era devices^31,37,38^. Substituting the encoding from (15) into (14), we obtain an approximate QUBO Hamiltonian:

16 $$\begin{aligned} H(q) = \sum _{i=1}^N \sum _{r=1}^R \alpha _i^r q_i^r + \sum _{i,j=1}^N \sum _{r,s=1}^R \beta _{ij}^{rs} q_i^r q_j^s, \end{aligned}$$

with coefficients

17 $$\begin{aligned} \alpha _i^r = -2^{-r+1} \left( \sum _{j=1}^N \sum _{k=1}^N A_{ki} A_{kj} c_i d_j + \sum _{j=1}^N A_{ji} c_i b_j \right) , \end{aligned}$$

18 $$\begin{aligned} \beta _{ij}^{rs} = 2^{-(r+s)} \left( \sum _{k=1}^N A_{ki} A_{kj} c_i c_j \right) . \end{aligned}$$

These expressions ensure that the binary-encoded variables minimize a cost function equivalent to the least-squares error of the original problem.

In the following, the algorithm of quantum annealing process for solving a linear system of equations is given:

**Algorithm 3:**
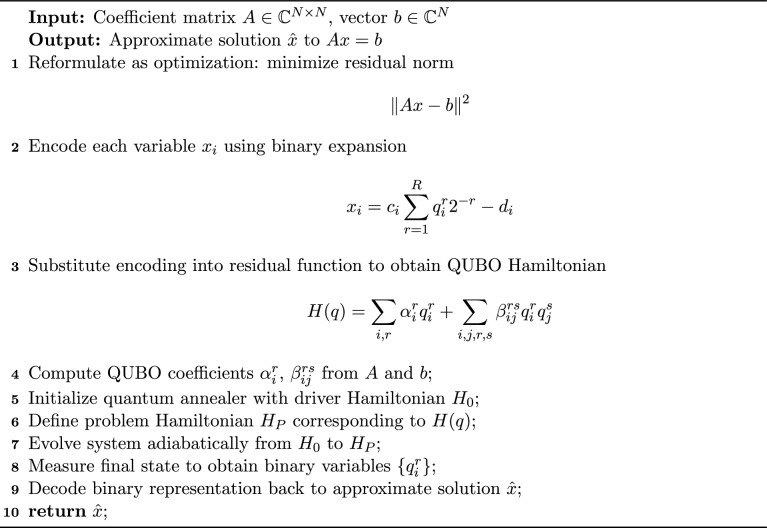
Quantum annealing process for solving linear system via QUBO

### Numerical example with quantum annealing

Consider again the linear systems obtained from the WEB-spline finite element discretization. In the following, we give the step by step of the algorithm:

**Step 1: Least-squares objective:** Solve $A x = b$ by minimizing the squared residual

$$x_{\textrm{sol}}=\arg \min _{x\in \mathbb {R}^4}\ \Vert A x-b\Vert _2^2 =\arg \min _x (A x-b)^\dagger (A x-b).$$

By writing $G:=A^T A$ and $h:=-2A^T b$, we have $\Vert A x-b\Vert _2^2 = x^T G x + h^T x + b^T b$.

**Step 2: Binary encoding of real variables:** For each component $x_i$, choose a bit-depth $R\in \mathbb {N}$, a scale $c_i>0$, and a shift $d_i$ and encode:

19 $$\begin{aligned} x_i = c_i \sum _{r=1}^{R} q_i^{\,r}\,2^{-r} - d_i,\qquad q_i^{\,r}\in \{0,1\}. \end{aligned}$$

Taking $d_i>0$ and $c_i>d_i/2$ allows signed ranges while limiting qubit count.

**Step 3: QUBO form:** Substitute (19) into the objective to obtain a quadratic function of the binary variables:

20 $$\begin{aligned} H(q) = \sum _{i=1}^{N}\sum _{r=1}^{R} \alpha _i^{\,r}\,q_i^{\,r} \;+\; \sum _{i,j=1}^{N}\sum _{r,s=1}^{R} \beta _{ij}^{\,rs}\,q_i^{\,r}q_j^{\,s} \;+\;\text {const}, \end{aligned}$$

with coefficients

$$\begin{aligned} \alpha _i^{\,r}&= -2^{-r+1}\!\left( \sum _{j=1}^{N}\sum _{k=1}^{N} A_{k i}A_{k j}\,c_i d_j \;+\; \sum _{j=1}^{N} A_{j i}\,c_i b_j \right) , \\ \beta _{ij}^{\,rs}&= 2^{-(r+s)} \left( \sum _{k=1}^{N} A_{k i}A_{k j}\,c_i c_j\right) . \end{aligned}$$

(An equivalent construction is obtained by first expanding $x^T G x + h^T x$ and then collecting linear and quadratic binary terms.)

**Step 4: Optional Ising mapping:** If the hardware uses Ising spins $s\in \{-1,+1\}$, apply $q=(s+1)/2$ to map (20) to $H(s)=\sum _\ell h_\ell s_\ell +\sum _{\ell <m} J_{\ell m} s_\ell s_m + \text {const}$ and compute fields/couplings $\{h_\ell ,J_{\ell m}\}$.

**Step 5: Embedding and annealing.** Minor-embed the QUBO/Ising graph on the device topology, choose anneal schedule and time, run multiple reads and obtain a set of bitstrings $q^\star$ minimizing (20).

**Step 6: Decoding to real solution:** Convert the best bitstring back to a real vector via

$$\hat{x}_i = c_i \sum _{r=1}^{R} q_i^{\star \,r}\,2^{-r} - d_i,\qquad i=1,\dots ,N.$$

**Step 7: Verification:** Evaluate the residual $\rho =\Vert A\hat{x}-b\Vert _2$. If $\rho$ exceeds tolerance, adjust $(R,c_i,d_i)$, rescale, or apply a brief classical refinement initialized at $\hat{x}$.

The resulting QUBO problem is solved using the simulated annealing solver available in the D-Wave Ocean SDK. Simulated annealing is a classical metaheuristic inspired by the physical process of cooling metals, where the system gradually lowers its energy state by probabilistically accepting both improving and, with decreasing likelihood, worsening moves. Within the Ocean framework, the QUBO objective function derived from the linear system is provided as input to the solver, which then generates candidate solutions by iteratively exploring the binary configuration space. By controlling parameters such as the number of sweeps and the annealing schedule, the solver balances exploration and exploitation to avoid local minima and approximate the global optimum. The best configuration found is subsequently mapped back into the real-valued solution space, providing an approximate solution vector to the original linear system.

Consequently, the quantum annealing algorithm enables the solution of the linear system derived from the proposed method. The computed solution is given in Table 7. The quantum annealing results in Table 7 report runtimes of up to approximately 3475s, which capture the complete simulated annealing workflow. This includes the construction and encoding of the QUBO formulation, the execution of many annealing sweeps and sampling cycles and the subsequent classical post-processing required to decode the approximate solution. As with VQLS, these values represent full end-to-end wall-clock times.

**Table 7:** Quantum annealing performance for WEBFEM systems.

*h*	Size	*R*	Qubits	$\Vert A \hat{x} - b\Vert$	Time (s)
0.5	$4\times 4$	2	8	$2.3159\times 10^{-2}$	44.8760
0.5	$4\times 4$	3	12	$2.1601\times 10^{-2}$	96.5716
0.5	$4\times 4$	4	16	$2.1848\times 10^{-2}$	159.0780
0.25	$13\times 13$	2	26	$1.9852\times 10^{-2}$	426.7425
0.25	$13\times 13$	3	39	$1.2864\times 10^{-2}$	916.1787
0.25	$13\times 13$	4	52	$1.0853\times 10^{-2}$	1676.7349
0.2	$19\times 19$	2	38	$2.5906\times 10^{-2}$	885.5661
0.2	$19\times 19$	3	57	$2.3492\times 10^{-2}$	1950.2081
0.2	$19\times 19$	4	76	$2.1760\times 10^{-2}$	3475.3674

**Figure 12:**
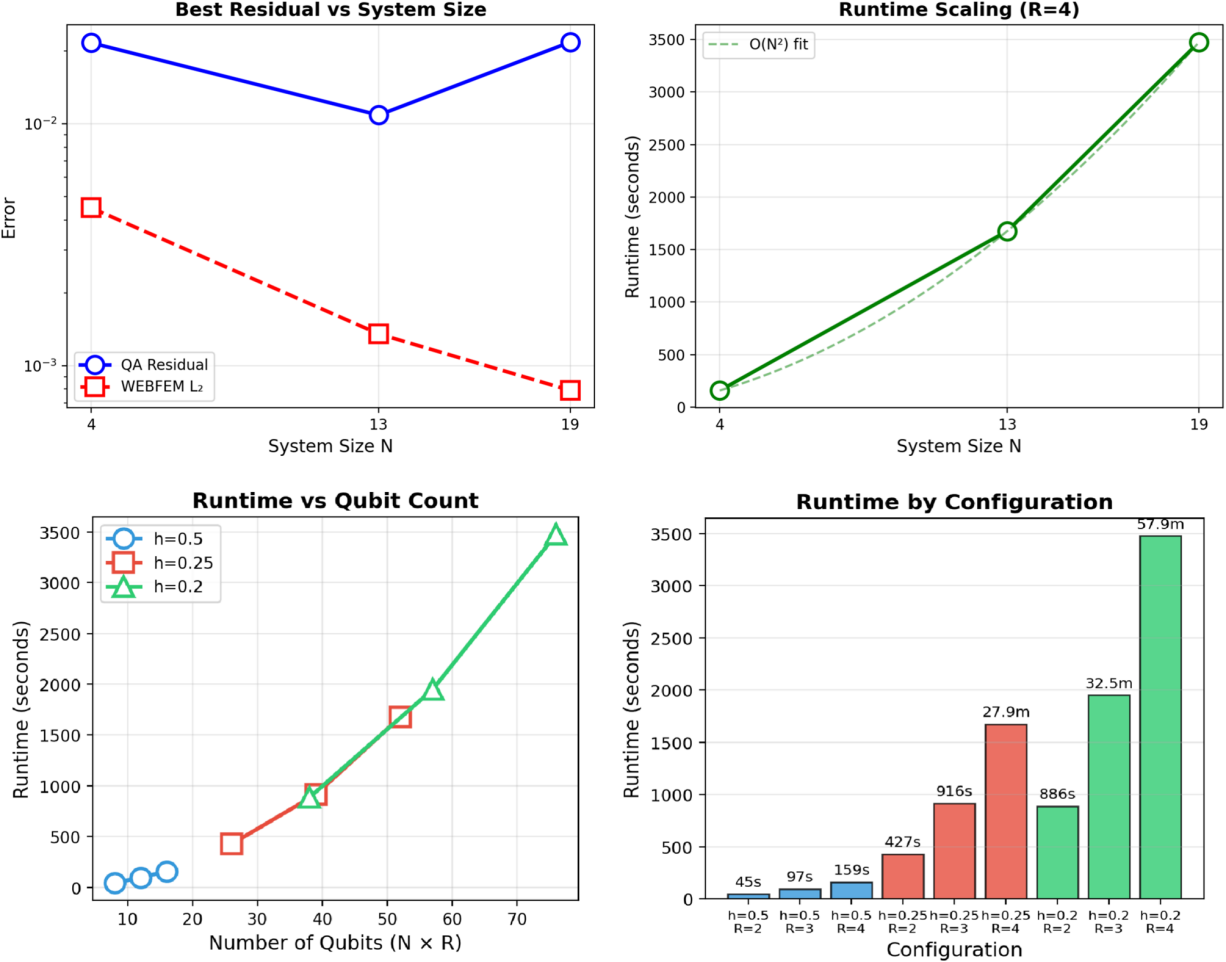
QA algorithm analysis for WEBFEM.

Figure 12 summarizes the scaling behavior of the QA solver for WEBFEM systems. The best residuals remain roughly constant at $\mathcal {O}(10^{-2})$ across all system sizes, whereas the WEBFEM $L_{2}$ error decreases with mesh refinement, indicating that QA accuracy does not improve with problem resolution. Runtime, however, increases sharply with system size, following an empirical $O(N^{2})$ trend, with the largest system exceeding 3300 seconds for $R=4$. When plotted against the total qubit requirement ($N \times R$), runtime grows approximately linearly, showing that embedding size is the dominant cost driver. Configuration-wise comparisons further illustrate how increasing both system dimension and repetition number compounds the runtime burden, with the $(h=0.2, R=4)$ case approaching one hour. Overall, QA residuals remain stable across scales, while runtime grows steeply with both problem size and qubit resources.

## Conclusion and comparison between VQLS, HHL, and quantum annealing

Solving linear system of equations is a foundational problem in computational science and engineering. Classical methods like Gaussian elimination scale poorly for large systems, motivating quantum approaches. The VQLS, HHL algorithm and quantum annealing represent three distinct paradigms leveraging quantum resources to accelerate or approximate linear system solutions. Each approach differs in computational model, circuit requirements and suitability for NISQ-era devices^7–9^.

VQLS is a hybrid quantum-classical method that uses a parameterized quantum circuit to represent the solution vector $|x\rangle$. The algorithm minimizes a cost function, such as $\Vert A|x\rangle - |b\rangle \Vert ^2$, using a classical optimizer. Its key strength is compatibility with near-term noisy devices, as it avoids deep circuits and complex subroutines like phase estimation. However, the performance depends heavily on the ansatz choice, optimizer efficiency and initialization^7^.

HHL is a circuit-based quantum algorithm that directly encodes the solution vector as a quantum state $|x\rangle = A^{-1}|b\rangle$. By employing quantum phase estimation (QPE) and controlled rotations, it effectively inverts the eigenvalues of $A$ to construct the solution state. In theory, HHL provides an exponential speedup over classical dense matrix inversion, with runtime scaling as $\mathcal {O}(\log N\,\kappa ^2 s^2/\epsilon )$, where $N$ is the system size, $\kappa$ is the condition number of $A$, $\epsilon$ is the desired precision and $s$ denotes the sparsity. The efficiency of HHL is therefore guaranteed only for sparse, well-conditioned matrices. A major limitation is the requirement of long, coherent quantum circuits and high-fidelity QPE, which restricts its practical implementation on current NISQ hardware^8^.

Quantum annealing maps the linear system to an optimization problem, typically minimizing $\Vert Ax-b\Vert ^2$, and encodes the solution in a QUBO or Ising Hamiltonian. The annealer then probabilistically searches for the ground state. QA is hardware-friendly for NISQ devices and allows parallel exploration of the solution space. However, it provides an approximate solution and may require repeated sampling, post-processing, or classical refinement to reach high accuracy^9^.

VQLS circuits are shallow but require repeated measurements and classical optimization loops. HHL circuits are deeper due to QPE and controlled rotations, potentially scaling poorly with system size and condition number. QA requires no traditional quantum circuits but depends on the annealer’s connectivity and qubit count. In terms of depth, VQLS is the most practical for near-term hardware, HHL is resource-intensive and QA offloads complexity to the annealing process^7–9^.

HHL theoretically provides exponential speedup for well-conditioned, sparse matrices. VQLS provides polynomial scaling advantages for certain ansatze but lacks guaranteed exponential speedup. QA’s performance depends on problem embedding and annealing schedule; for large dense systems, qubit requirements and minor embedding overhead may reduce its effective speedup. In practical terms, QA and VQLS are more suitable for moderate-sized problems on NISQ hardware^7–9^.

VQLS is robust to certain noise levels because the variational approach can adapt to noisy measurements through the optimizer. HHL is highly sensitive to gate errors, decoherence and QPE inaccuracies, as small errors in phase estimation directly affect eigenvalue inversion. QA is resilient to moderate thermal noise due to the probabilistic nature of annealing but can suffer from embedding errors and limited precision in representing real-valued solutions^7–9^.

Both HHL and VQLS produce a quantum state representing the solution $|x\rangle$, from which only expectation values or specific observables can be extracted efficiently. In principle, full reconstruction of the solution vector would require quantum state tomography, which scales exponentially with the system size and is therefore infeasible for large problems (see Appendix ??). In HHL, tomography or advanced measurement protocols such as swap tests are necessary to recover amplitudes or specific entries of $|x\rangle$. VQLS, while still outputting a quantum state, allows more flexible extraction: tomography can be applied, but often expectation values are sufficient and classical post-processing can be leveraged due to the ansatz structure. In contrast, quantum annealing naturally produces classical bitstrings corresponding to low-energy solutions of the QUBO problem; these bitstrings can be directly decoded into approximate real-valued solutions, avoiding the need for tomography altogether^7–9^.

VQLS is ideal for NISQ devices due to shallow circuits and iterative optimization. HHL is best suited for fault-tolerant quantum computers because it relies on deep circuits and precise phase estimation. QA benefits from specialized quantum annealers (e.g., D-Wave), with native support for binary optimization, limited connectivity, and thermal noise. The choice between methods often depends on available hardware, desired precision, and problem size^7–9^.

**Figure 13:**
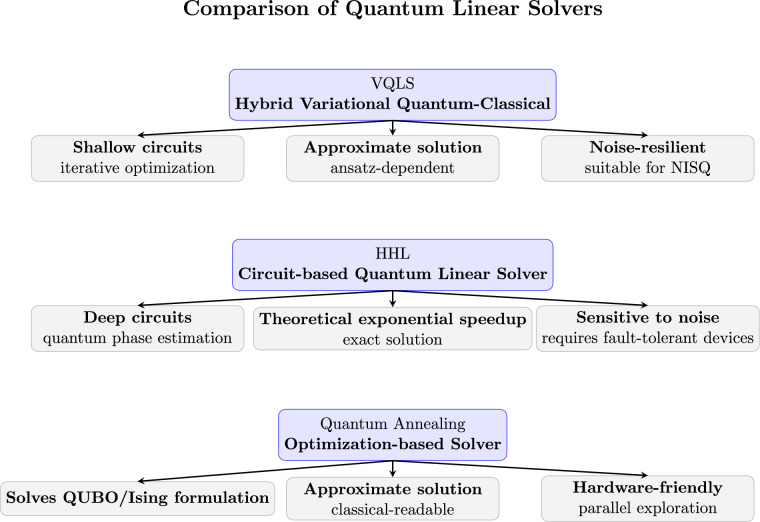
Comparison of VQLS, HHL, and quantum annealing highlighting circuit depth, solution type, and hardware suitability.

In summary, VQLS offers flexibility and near-term feasibility with approximate solutions, HHL provides theoretically exact solutions with exponential speedup but is hardware demanding and QA delivers classical-readable approximate solutions efficiently on annealers, particularly for structured or sparse problems. The selection among these methods depends on the trade-off between solution accuracy, circuit depth, hardware availability, and problem scale, making them complementary tools in quantum linear algebra^7–9^. In Figure 13 a comparison of VQLS, HHL, and quantum annealing is given in brief.

## Supplementary Information

Supplementary Information.

## Data Availability

The datasets used and/or analysed during the current study available from the corresponding author on reasonable request.
