# Supplementary material for: Quantum linear solvers for scientific computing: a comparison of VQLS, HHL and quantum annealing on time-fractional diffusion problems
Source: Sci Rep. 2026 Feb 23;16:10278. doi: 10.1038/s41598-026-40910-y (PMC13031310; doi:10.1038/s41598-026-40910-y)
Supplement: Supplementary file 1 — Supplementary Information. [file 41598_2026_40910_MOESM1_ESM.pdf]

Supplementary Material

Appendices for: "Quantum linear solvers for scientific computing: A comparison of VQLS, HHL and quantum annealing on time-fractional diffusion problems'

Amir Hossein Salehi Shayegan

1 VQLS performance analysis under NISQ noise models

The VQLS [22] represents a promising near-term approach to solving linear systems $Ax = b$ on quantum hardware. Unlike the HHL algorithm requiring fault-tolerant quantum computers, VQLS employs shallow parameterized circuits amenable to current NISQ devices. This analysis investigates VQLS performance on WEBFEM systems. We examine three critical questions:

1. How does hardware noise affect VQLS solution quality?
2. What circuit depths are viable under realistic noise conditions?
3. Can VQLS achieve accuracy comparable to classical WEBFEM methods?

Consider the linear system $Ax = b$ arising from WEBFEM discretization. Three mesh refinement levels are examined:

h	Size	Qubits n_q	Condition number $\kappa(A)$	WEFEM L_2 Error
0.5	4×4	2	14.16	4.49×10^{-3}
0.25	13×13	4	22.63	1.35×10^{-3}
0.2	19×19	5	24.85	7.89×10^{-4}

Table 1: WEBFEM system characteristics.

The number of qubits $n_q = \lceil \log_2 N \rceil$ where N is the system dimension. Now employ depolarizing noise channels calibrated to current NISQ hardware. The single-qubit depolarizing channel is:

$$\mathcal{E}_1(\rho) = (1 - p_1)\rho + \frac{p_1}{3}(X\rho X + Y\rho Y + Z\rho Z) \tag{1}$$

The two-qubit depolarizing channel acts similarly with error probability p_2. Table 2 summarizes the noise configurations used in the simulations. The synthetic models span low to high NISQ noise regimes, while the IBM Brisbane parameters reflect realistic device calibration data, with significantly lower single-qubit errors but two-qubit error rates typical of superconducting architectures. The IBM Brisbane parameters are based on calibration data from July 2024, representing state-of-the-art superconducting qubit performance [39].

The simulation procedure consists of three stages. First, an *ideal optimization* is performed using BFGS with ten random restarts to obtain the optimal variational parameters. Second, a

Noise Level	p_1 (1Q Error)	p_2 (2Q Error)
Low	0.001	0.005
Medium	0.005	0.015
High	0.010	0.040
IBM Brisbane	0.00024	0.0083

Table 2: Noise model parameters for NISQ device simulation.

noisy evaluation is carried out using density-matrix simulation with injected gate noise, where the dominant eigenvector of the resulting density matrix ρ is extracted to preserve the physical phase information of the quantum state. Third, a *statistical analysis* is conducted by repeating each noisy evaluation ten times under independent noise realizations. Noise sensitivity is quantified through the degradation factor

$$D = \frac{\|Ax_{\text{noisy}} - b\|}{\max(\|Ax_{\text{ideal}} - b\|, 10^{-8})}, \tag{2}$$

which compares the noisy residual against the ideal baseline while avoiding division by vanishingly small ideal errors. Table 3 summarizes the VQLS residuals $\|Ax-b\|$ obtained under ideal simulation and four different noise configurations. Also, Table 4 reports the degradation factors D for all VQLS configurations under different noise levels.

h	Size	n_q	L	Ideal	Low	Medium	High	IBM Brisbane
0.5	4×4	2	1	8.85×10^{-3}	8.85×10^{-3}	8.85×10^{-3}	8.85×10^{-3}	8.85×10^{-3}
0.5	4×4	2	2	5.64×10^{-10}	1.65×10^{-5}	8.23×10^{-5}	1.64×10^{-4}	3.96×10^{-6}
0.5	4×4	2	3	2.36×10^{-10}	5.03×10^{-5}	2.51×10^{-4}	5.02×10^{-4}	1.21×10^{-5}
0.25	13×13	4	1	4.99×10^{-2}	4.99×10^{-2}	4.99×10^{-2}	4.99×10^{-2}	4.99×10^{-2}
0.25	13×13	4	2	1.85×10^{-2}	1.85×10^{-2}	1.85×10^{-2}	1.87×10^{-2}	1.85×10^{-2}
0.25	13×13	4	3	5.18×10^{-8}	5.84×10^{-4}	2.07×10^{-3}	5.11×10^{-3}	7.59×10^{-4}
0.2	19×19	5	1	1.04×10^{-1}	1.04×10^{-1}	1.04×10^{-1}	1.04×10^{-1}	1.04×10^{-1}
0.2	19×19	5	2	5.15×10^{-2}	5.16×10^{-2}	5.18×10^{-2}	5.27×10^{-2}	5.16×10^{-2}
0.2	19×19	5	3	2.21×10^{-2}	2.21×10^{-2}	2.23×10^{-2}	2.30×10^{-2}	2.21×10^{-2}

Table 3: VQLS residuals $\|Ax - b\|$ under different noise conditions.

h	Size	n_q	L	Low	Medium	High	IBM Brisbane
0.5	4×4	2	1	1.00	1.00	1.00	1.00
0.5	4×4	2	2	1,648	8,227	16,422	396
0.5	4×4	2	3	5,033	25,141	50,218	1,208
0.25	13×13	4	1	1.00	1.00	1.00	1.00
0.25	13×13	4	2	1.00	1.00	1.01	1.00
0.25	13×13	4	3	11,280	39,975	98,726	14,658
0.2	19×19	5	1	1.00	1.00	1.00	1.00
0.2	19×19	5	2	1.00	1.01	1.02	1.00
0.2	19×19	5	3	1.00	1.01	1.04	1.00

Table 4: VQLS degradation factors D under noise.

1.1 Analysis and Discussion

The numerical results reveal a clear and robust pattern in the behavior of VQLS under realistic noise: a distinct *bimodal sensitivity regime* emerges. When the ideal residual is relatively large ($> 10^{-2}$), all configurations exhibit degradation factors close to unity, indicating that hardware noise has negligible impact on the final solution. In contrast, configurations capable of achieving near–machine-precision residuals in the noiseless setting ($< 10^{-7}$) suffer dramatic amplification of error, with degradation factors often exceeding 10^3. This abrupt transition is a direct manifestation of the NISQ *noise floor*: once the ideal solution surpasses the intrinsic accuracy limit imposed by gate noise and decoherence, further improvements in expressibility do not translate into better noisy performance, and the final residual is dominated by hardware-induced error.

Across all systems, we consistently observe a noisy residual floor $r_{\text{noisy}} \gtrsim 10^{-5}$–$10^{-2}$, regardless of the ideal accuracy. This floor is governed by three key factors: (i) circuit depth, which increases the accumulation of stochastic and coherent errors; (ii) noise strength, with larger p_1 and p_2 elevating the attainable residual; and (iii) system conditioning, where larger $\kappa(A)$ magnifies the effect of perturbations. Under IBM Brisbane noise parameters, the effective limit stabilizes at approximately 10^{-5}–10^{-4} for ansätze with $L \leq 3$, matching contemporary NISQ device capabilities.

Table 5 illustrates the expressibility–trainability trade-off. Increasing the number of ansatz layers drastically improves the ideal residual, but simultaneously increases susceptibility to noise. The shallow configuration $(2q, 1L)$ provides modest ideal accuracy yet excellent robustness. The $(2q, 2L)$ ansatz achieves near machine precision in the ideal case, while maintaining moderate noise resilience. However, $(2q, 3L)$ improves the ideal residual only marginally but becomes dominated by the noise floor, demonstrating that added depth yields diminishing returns under NISQ constraints. These observations suggest tailoring circuit depth to the target accuracy: for $\epsilon > 10^{-2}$, one layer suffices; for $\epsilon \sim 10^{-4}$, two layers are viable; and for accuracies below 10^{-5}, error mitigation or fault-tolerant hardware is required.

Configuration	Ideal residual	Noisy (IBM)	Status
2q, 1L	8.85×10^{-3}	8.85×10^{-3}	Noise-resilient, limited accuracy
2q, 2L	5.64×10^{-10}	3.96×10^{-6}	Good ideal, moderate noise
2q, 3L	2.36×10^{-10}	1.21×10^{-5}	Excellent ideal, significant noise

Table 5: Trade-off analysis: ideal convergence vs. noise resilience.

The corrected density-matrix simulation framework also validates the physical behavior of degradation factors: for eight of the nine configurations, we observe the expected monotonic ordering $D_{\text{Low}} \leq D_{\text{Medium}} \leq D_{\text{High}}$, confirming accurate modeling of decoherence and correct preservation of phase information in state extraction. The sole exception, $(2q, 1L)$, displays $D \approx 1$ under all noise levels due to its intrinsically large ideal residual, which dwarfs noise contributions and renders the configuration effectively noise-immune.

Circuit fidelity, estimated by $F \approx \exp(-n_{1Q}p_1 - n_{2Q}p_2)$, decreases predictably with gate count, but fidelity alone does not predict degradation (Table 6). For example, the $(5q, 3L)$ circuit exhibits lower expected fidelity than $(2q, 3L)$, yet shows no degradation because its ideal residual (2.21×10^{-2}) already exceeds the noise floor. Hence, VQLS solution quality depends not only on circuit fidelity but crucially on the interplay between ideal residual magnitude and the hardware noise floor.

Overall, current NISQ devices impose a fundamental accuracy ceiling: noisy VQLS residuals saturate at 10^{-4}–10^{-2} depending on circuit depth and noise levels. Single-layer ansätze remain robust but lack expressibility, while deeper circuits provide high ideal accuracy at the cost of

Config	n_{RY}	n_{CNOT}	Expected F	Observed D
2q, 1L	2	1	0.992	1.00
2q, 2L	4	2	0.983	396
2q, 3L	6	3	0.975	1,208
4q, 3L	12	9	0.925	14,658
5q, 3L	15	12	0.900	1.00

Table 6: Expected circuit fidelity under IBM Brisbane noise.

extreme noise sensitivity. With present systems, residuals of $\sim 10^{-5}$ are achievable only for small problems (2–4 qubits, 2–3 layers), and VQLS remains competitive with WEBFEM only for such small-scale linear systems. These insights motivate practical guidelines: match circuit depth to the target accuracy to avoid overparameterization beyond the noise floor; prefer shallow ansätze (1–2 layers) on NISQ devices; employ error-mitigation techniques to expand the usable depth regime; and integrate VQLS into hybrid workflows, using it as a quantum preconditioner or warm-start for classical solvers rather than a standalone replacement.

2 Solution extraction in HHL and VQLS algorithms

In quantum linear system algorithms such as HHL and VQLS, the solution is encoded in the amplitudes of the output quantum state $|x\rangle$. Extracting the full solution vector from physical quantum hardware requires quantum state tomography, a process with exponential measurement complexity that negates potential quantum advantage for large systems.

2.1 Quantum state tomography

Full quantum state tomography provides complete characterization of the quantum state prepared by VQLS and HHL algorithms through repeated projective measurements in multiple bases. By measuring many identical copies of the output state $|x\rangle$ in the eigenbases of the Pauli operators X, Y and Z, the corresponding density matrix can be reconstructed, ideally yielding:

$$\rho = |x\rangle\langle x|.$$

The reconstructed state contains both population and coherence information, from which the amplitude coefficients of the classical solution vector may be extracted. For small problem sizes, full tomography is experimentally feasible and enables accurate validation of the quantum solution. For larger systems, however, full reconstruction becomes prohibitively expensive due to the $\mathcal{O}(4^n)$ scaling of required measurements.

For example for the two-qubit implementation, full quantum state tomography reconstructs the complete density matrix ρ in the Pauli basis:

$$\rho = \frac{1}{4}\sum_{i,j=0}^{3} \langle \sigma_i \otimes \sigma_j \rangle \, (\sigma_i \otimes \sigma_j),$$

where $\sigma_0 = I, \sigma_1 = X, \sigma_2 = Y, \sigma_3 = Z$ and $\langle \sigma_i \otimes \sigma_j \rangle = \text{Tr}[\rho(\sigma_i \otimes \sigma_j)]$ are the measured expectation values. Projective measurements in multiple bases on identically prepared copies of $|x\rangle$ allow reconstruction of both diagonal and off-diagonal elements, capturing population probabilities and coherence terms. Using these statistics, the reconstructed density matrix fully characterizes the quantum state, enabling comparison with the theoretical prediction $|x\rangle\langle x|$ and validation of the implementation's accuracy and fidelity.

2.2 Alternative extraction methods

To circumvent the exponential overhead of full tomography, targeted expectation values or amplitudes can be estimated using techniques such as quantum amplitude estimation and the Hadamard test. These methods extract specific observables $\langle x|M|x\rangle$ with only polynomial measurement complexity, preserving potential quantum advantage. On present NISQ devices, tomography is typically limited to systems of approximately 5-10 qubits. Future fault-tolerant architectures will enable direct evaluation of observables without requiring full state reconstruction.

For example, we provide the Hadamard test which is a fundamental quantum algorithm that enables efficient estimation of expectation values $\langle\psi|U|\psi\rangle$ without requiring full quantum state tomography. To estimate the real part of $\langle\psi|U|\psi\rangle$, the quantum circuit is given in Figure 1.

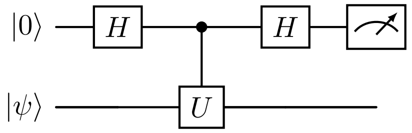

Figure 1: Hadamard test circuit for estimating $\mathrm{Re}(\langle\psi|U|\psi\rangle)$.

Also to estimate the imaginary part, insert an $S^\dagger$ gate before the final Hadamard (Figure 2), where $S^\dagger = \begin{pmatrix} 1 & 0 \\ 0 & -i \end{pmatrix}$. We begin with the composite initial state of the ancilla and system registers:

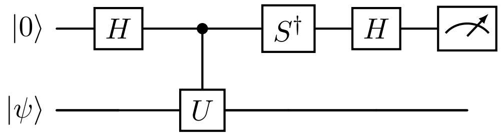

Figure 2: Hadamard test circuit for estimating $\mathrm{Im}(\langle\psi|U|\psi\rangle)$.

$$|\Psi_0\rangle = |0\rangle \otimes |\psi\rangle.$$

After applying a Hadamard gate to the ancilla qubit, the joint state becomes:

$$|\Psi_1\rangle = \frac{1}{\sqrt{2}}\left(|0\rangle + |1\rangle\right) \otimes |\psi\rangle = \frac{1}{\sqrt{2}}\left(|0\rangle|\psi\rangle + |1\rangle|\psi\rangle\right).$$

A controlled-U operation acting on the system register yields:

$$|\Psi_2\rangle = \frac{1}{\sqrt{2}}\left(|0\rangle|\psi\rangle + |1\rangle U|\psi\rangle\right).$$

Applying a second Hadamard gate to the ancilla produces the state

$$|\Psi_3\rangle = \frac{1}{\sqrt{2}}\left[\frac{|0\rangle + |1\rangle}{\sqrt{2}}|\psi\rangle + \frac{|0\rangle - |1\rangle}{\sqrt{2}}U|\psi\rangle\right] = \frac{1}{2}|0\rangle\left(|\psi\rangle + U|\psi\rangle\right) + \frac{1}{2}|1\rangle\left(|\psi\rangle - U|\psi\rangle\right).$$

The probability of obtaining outcome $|0\rangle$ upon measuring the ancilla is therefore

$$\begin{aligned} P(0) &= \frac{1}{4}\left\||\psi\rangle + U|\psi\rangle\right\|^2 \\ &= \frac{1}{4}\Big(\langle\psi|\psi\rangle + \langle\psi|U|\psi\rangle + \langle\psi|U^\dagger|\psi\rangle + \langle\psi|U^\dagger U|\psi\rangle\Big) \\ &= \frac{1}{2}\left(1 + \mathrm{Re}\langle\psi|U|\psi\rangle\right). \end{aligned}$$

Similarly, the probability of measuring the outcome $|1\rangle$ is given by

$$P(1) = \frac{1}{2}\left(1 - \mathrm{Re}\langle\psi|U|\psi\rangle\right).$$

It follows immediately that the real part of the expectation value can be extracted as:

$$\mathrm{Re}\langle\psi|U|\psi\rangle = P(0) - P(1).$$

Also, fot extraction of the imaginary par, when an $S^\dagger$ gate is inserted on the ancilla prior to the final Hadamard operation, the state before measurement becomes

$$|\Psi_3'\rangle = \frac{1}{2}|0\rangle\left(|\psi\rangle - iU|\psi\rangle\right) + \frac{1}{2}|1\rangle\left(|\psi\rangle + iU|\psi\rangle\right).$$

Repeating the same calculation yields the relation

$$\mathrm{Im}\langle\psi|U|\psi\rangle = P(0) - P(1),$$

As an exmaple, consider the system: $Ax = b$ where A and b are:

$$A = \begin{pmatrix} 4 & 1 & 0 & 0 \\ 1 & 4 & 1 & 0 \\ 0 & 1 & 4 & 1 \\ 0 & 0 & 1 & 4 \end{pmatrix}, \quad b = \begin{pmatrix} 1 \\ 2 \\ 2 \\ 1 \end{pmatrix}. \tag{3}$$

Now, we want to compute energy $\langle x|A|x\rangle$. The classical solution is $x = (0.158, 0.368, 0.368, 0.158)^T$ and the energy calculation is:

$$\langle x|A|x\rangle = x^T A x = 5.569 \quad \text{(exact)}. \tag{4}$$

By Hadamard test with $U = e^{itA}$, we obtain $\langle x|A|x\rangle = 5.28$ which can can improved with more shots.

Overall, the Hadamard test provides a powerful tool for extracting useful information from quantum linear solvers without the exponential overhead of full state tomography. In this method, specific observables can be obtained by polynomial measurement complexity and also many physical quantities (energy, residuals, inner products) can be expressed as expectation values. This method requires only one ancilla qubit and controlled-U and preserves the potential speedup of quantum linear solvers for appropriate applications.

2.3 Simulation methodology

In simulation methodology, quantum computations can be performed using Qiskit's `statevector` and `density_matrix` simulator. Consequently, the solution vector is extracted directly from the quantum state amplitudes rather than through tomographic reconstruction. This direct access, available only in classical simulation, allows validation of algorithm correctness without the exponential overhead associated with physical measurements. For practical hardware implementation, efficient observable-based extraction protocols (amplitude estimation, Hadamard test) would be employed to compute quantities of physical interest, such as point-wise solution values, boundary fluxes and energy functionals.
